# In Situ Electron Tomography Insights into the Curvature Effect of a Concave Surface on Fe Single Atoms for Durable Oxygen Reaction

**DOI:** 10.1002/advs.202412387

**Published:** 2024-12-16

**Authors:** Jun‐Kang Li, Haobo Zhao, Yang Zhang, Jing‐Jing Ma, Fen‐Fen Wang, Shu‐Na Zhao, Jun Li, Shuang‐Quan Zang

**Affiliations:** ^1^ Henan Key Laboratory of Crystalline Molecular Functional Materials College of Chemistry and Pingyuan Laboratory Zhengzhou University Zhengzhou 450001 China; ^2^ Department of Chemistry and Key Laboratory of Organic Optoelectronics and Molecular Engineering of Ministry of Education Tsinghua University Beijing 100084 China; ^3^ School of Materials Science and Engineering Center of Advanced Analysis and Gene Sequencing Zhengzhou University Zhengzhou 450001 China; ^4^ Department of Chemistry and Engineering Research Center of Advanced Rare‐Earth Materials of Ministry of Education Tsinghua University Beijing 100084 China; ^5^ Fundamental Science Center of Rare Earths Ganjiang Innovation Academy Chinese Academy of Science Ganzhou 341000 China

**Keywords:** curvature effect, electron tomography, flexible Zn–air batteries, oxygen reduction reaction, single‐atom catalysts

## Abstract

Curvature‐induced interfacial electric field effects and local strain engineering offer a powerful approach for optimizing the intrinsic catalytic activity of single‐atom catalysts (SACs). Investigations into the surface curvature on SACs are still ongoing, and the impact of the concave surface is often overlooked. In this work, theoretical calculations indicate that curved surfaces, particularly those with concavity, can optimize the electronic structures of single Fe sites and facilitate the reductive release of *OH. A carbon sphere featuring uniformly oriented channels and a chiral multi‐shelled carbon hollow nanosphere are selected as carbon matrices due to their accessible concave and/or convex surfaces. After loading Fe species, the resulting catalysts with Fe SA in curved surfaces exhibit excellent oxygen reduction reaction activity (*E*_1/2_ = ≈0.89 V), strong methanol tolerance, and favorable long‐term stability. Impressively, a solid‐state flexible Zn–air battery based on this catalyst exhibits a remarkable durability over 40 h with a high peak power density of 122.1 mW cm^−2^ and excellent charge–discharge performance at different bending angles. This work offers in‐depth insights into the rational design of carbon supports with highly curved surfaces, offering new opportunities for the microenvironmental regulation of SACs at the atomic level.

## Graphical abstract

The concave surfaces of carbon matrices can influence the electronic structures of Fe─N─C sites, providing a powerful approach for optimizing the intrinsic catalytic activity of single‐atom catalysts. A solid‐state flexible ZAB based on Fe/CCNs‐P123 exhibits a remarkable durability of 40 h with a high peak power density of 122.1 mW cm^−2^ and excellent charge–discharge performance at different bending angles.

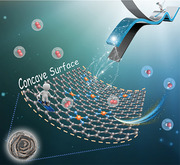

## 1 Introduction

Proton‐exchange membrane fuel cells (PEMFCs) hold great promise for generating clean electricity due to their high energy density, superior conversion efficiency, and zero carbon emissions.^[^ ^1^ ^]^ However, the widespread adoption of PEMFCs in automobile applications has been hindered by the utilization of scarce and expensive Pt‐group metal (PGM) catalysts required to accelerate the sluggish oxygen reduction reaction (ORR).^[^ ^2^ ^]^ Therefore, the rational design of PGM‐free catalysts with great activity and stability toward ORR is highly desired. With the merits of maximum atom‐utilization, tunable electronic environments, and strong resistance capacity to acidic/alkaline medium, carbon‐supported single‐atom catalysts (SACs) featuring M─N─C moieties have been experimentally and theoretically demonstrated as the most promising alternatives with outstanding ORR performance.^[^ ^3^ ^]^ Nevertheless, their catalytic performance is less satisfactory than those achieved by commercial cathode materials, highlighting the significance of further activity enhancement.

To this end, recent breakthroughs have focused on the strategic manipulation of the geometric and electronic structures to enhance the intrinsic activity of M─N─C sites, thereby optimizing absorption–desorption behavior of intermediate species.^[^ ^4^ ^]^ For example, heteroatom incorporation and axial coordination are widely employed strategies to enhance O_2_ adsorption ability and ORR performance of SACs by breaking the electron/geometric symmetry of M‐N_4_ sites.^[^ ^3^, ^5^ ^]^ Recently, dual‐metal–atom catalysts have emerged as a novel frontier for ORR, exhibiting enhanced reaction kinetics and reduced reaction barriers attributed to the d–d electronic interactions between adjacent isolated metal sites.^[^ ^6^ ^]^ However, all these M─N─C models are predicated on the assumption of an ideal carbon plane. The inherent curvature of the carbon matrix obtained by high‐temperature pyrolysis is usually overlooked.

In contrast to the plate structure, the surface curvature of the carbon matrix tends to generate an interfacial electric field and induce a local strain effect, potentially enhancing the catalytic activity.^[^ ^7^ ^]^ For example, Wang and coworkers demonstrated that the tip‐like FeN_4_ sites on spherical carbon surfaces generate a strong local electric field, which facilitates the accumulation of ORR‐related species (OH^–^, K^+^, and O_2_), thereby accelerating the ORR kinetics.^[^ ^7a^ ^]^ In addition, Lum and coworkers discovered that an increased nano‐curvature generates a stronger electric field, and this strategy is effective for various electrocatalytic reactions.^[^ ^7b^ ^]^ Currently, onion‐like carbons (OLCs) and carbon nanotubes (CNTs) with controlled diameters are the most commonly utilized carbon matrices for investigating the curvature effect.^[^ ^8^ ^]^ The introduction of surfactant micelles with ZIF precursors facilitates the formation of OLCs with high‐curvature surfaces, which may alter the adsorption/desorption energy of reaction intermediates and improve the catalytic performance of Fe SACs.^[^ ^8b^ ^]^ Recently, a helical carbon material featuring a plentiful curved carbon surface has been prepared by carbonization of well‐arranged helical polypyrrole induced by the chiral surfactants as a template.^[^ ^9^ ^]^ The high‐curvature surface induces a compressive strain effect on the FeN_4_ sites, resulting in an optimization of their electronic structure and a consequent reduction in the energy barrier for ORR. Despite the increasing attention drawn to the positive impact of surface curvature on the catalytic performance of SACs, current research mainly focuses on convex surfaces, with little mention of concave surfaces. Therefore, the design and synthesis of curved carbon matrices with accessible convex and concave surfaces are very crucial for the in‐depth investigation into the influence of curvature on the catalytic performance of SACs.

In this study, density functional theory (DFT) calculations demonstrate that the FeN_4_ sites on a curved surface, especially on the concave surface, can weaken the over‐strong adsorption of *OH and lower the energy barrier of the rate‐determining step (RDS), thus promoting the ORR kinetics. Although CNTs are the ideal model for investigating the impact of surface curvature on ORR, the study of the convex outer surface is relatively straightforward, whereas the analysis of the concave inner surface presents significant challenges. Surfactants, commonly employed as soft templates, not only facilitate the formation of carbon matrices with diverse pore structures but also promote surface curvature during the pyrolysis process.^[^ ^8b^ ^]^ A carbon sphere featuring abundant concave surfaces within the uniformly oriented channels was obtained by using Pluronic F127 as the soft template. By employing Pluronic P123, chiral curved carbon nanospheres (CCNs‐P123) were achieved, whose spiral architecture would induce the orientation of the carbon layers during the pyrolysis process. The abundant pore structures and surface curvatures of the two matrices are characterized by HRTEM and 3D reconstructions. The as‐obtained catalysts with Fe SA on a curved surface exhibit outstanding ORR performance (*E*_1/2_ = ≈0.89 V), surpassing that of the commercial Pt/C catalyst. Furthermore, a solid‐state flexible Zn–air battery constructed using Fe/CCNs‐P123 as the air electrode reveals a remarkable durability of 40 h with a high peak power density of 122.1 mW cm^−2^, and excellent charge–discharge performance at different bending angles.

## 2 Results and Discussion

**Figure 1:**
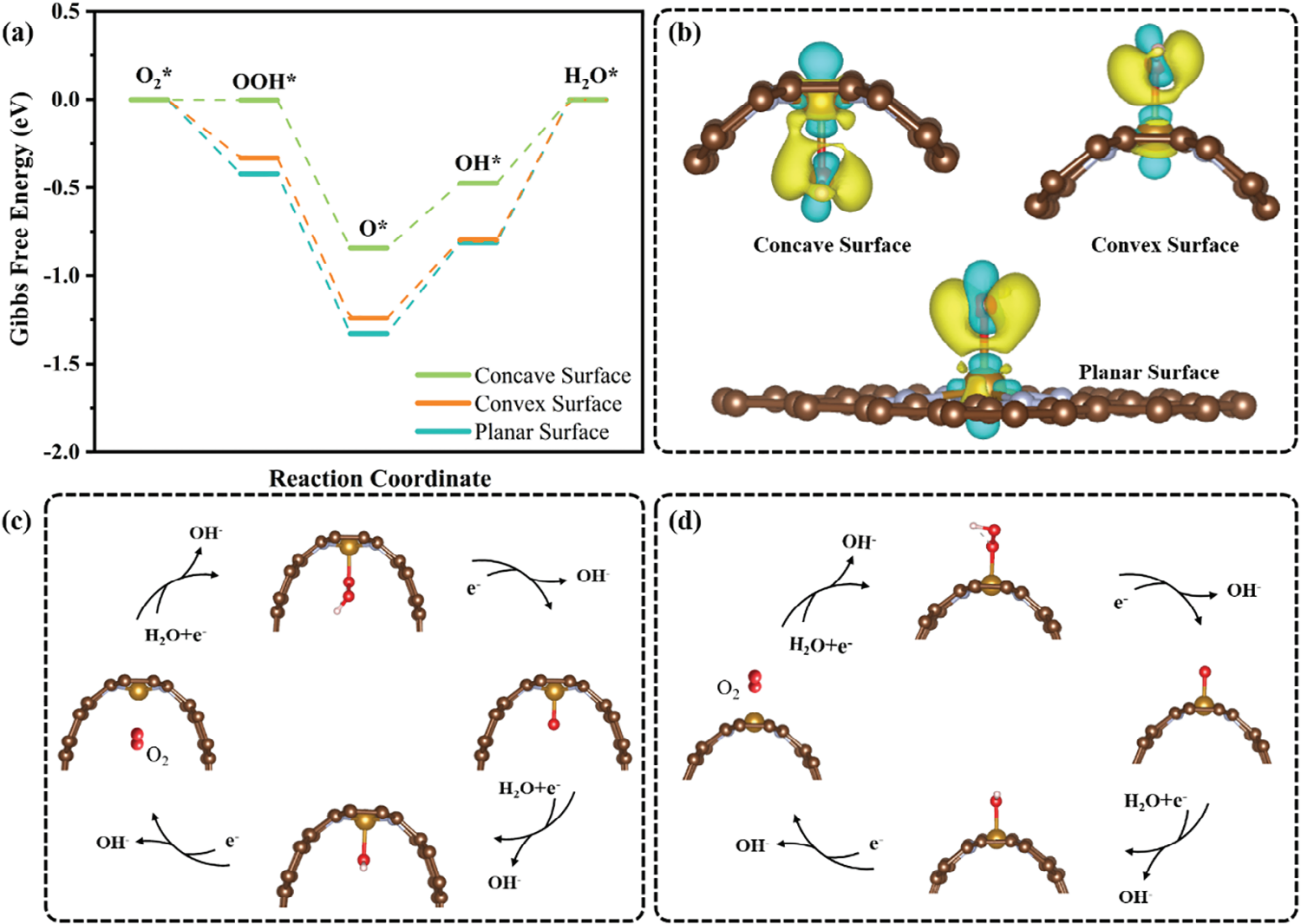
Theoretical calculations. a) Free energy diagrams of intermediates along the reaction coordinate at a potential of 1.23 V. b) Calculated charge density differences for concave, convex, and planar surfaces. The reaction schemes with the intermediates in the ORR process on c) concave and d) convex surfaces.

To elucidate the relationship between curved FeN_4_ sites (including both convex and concave surfaces) and ORR catalytic performance, comprehensive DFT calculations were first conducted. FeN_4_ sites in an armchair nanoribbon with a diameter of 10 Å or ∞ were established to model the surface curvature and flat structure, respectively. The free energy profiles of ORR processes on FeN_4_ sites in concave, convex, and flat surfaces were calculated at the potential of *U* = 1.23 V. As illustrated in **Figure** **1**a, the final *OH desorption step was identified as the RDS for all models, suggesting that the over strong adsorption of *OH on the Fe center might restrict the ORR performance. The ORR overpotentials of the Fe sites in concave and convex surfaces are 0.48 and 0.80 eV, respectively, both of which were lower than that of the Fe sites in flat surfaces (0.81 eV). This result suggests that the curved structure can facilitate *OH desorption and thus promote the ORR kinetics. Interestingly, the Fe sites in the concave surface show a significantly reduced overpotential compared to that in the convex surface, highlighting that the Fe sites in the concave surface make the most significant contribution to the excellent ORR performance of the catalyst. The charge density difference maps clearly indicate obvious charge transfer from the single Fe site to the absorbed *OH for both cases (Figure 1b). The bader charge analysis further confirms that electrons transferred from Fe sites in concave and convex surfaces to absorbed *OH are 0.40 and 0.43 e, respectively. Both values are lower than that in a flat surface (0.47 e), indicating that the curved surface, especially the concave surface, is advantageous for weakening the over‐strong adsorption of *OH on Fe sites and promoting the kinetics of ORR (Figure 1c,d, and Figure S1, Supporting Information). Furthermore, the crystal orbital Hamilton population (COHP) and integrated COHP analyses have been performed to evaluate the interaction between the Fe sites in concave/convex surfaces and the absorbed *OH. Quantitatively, the integrated bonding strength of Fe─*OH in convex surfaces was calculated to be −1.80 eV, which is negative than that of Fe─*OH in concave surfaces, resulting in the over‐strong Fe─*OH bonding strength, which agrees with the bader charge results (Figure S2, Supporting Information).

**Figure 2:**
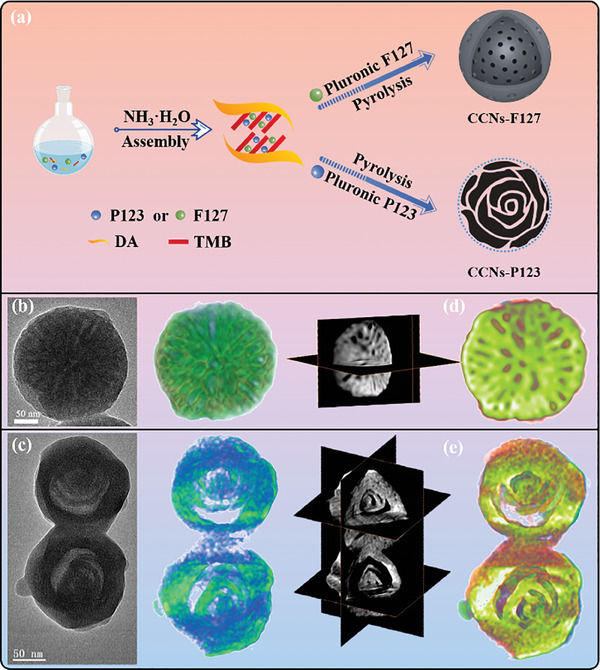
Fabrication route and morphological characterization of CCNs. a) Schematic illustration of the preparation of CCNs‐F127 and CCNs‐P123. Electron tomography reconstruction of b) CCNs‐F127 and c) CCNs‐P123 (before reaction). Left: TEM image; Middle: cross section view about reconstructed volume; Right: an overview of the orthoslice from different directions. The comparison of the cross section of d) CCNs‐F127 and e) CCNs‐P123 before and after electro‐catalysis reactions. Red color means sample before reaction, green color means after reaction. The 3D volume rendering images and 3D reconstructed slices show that the interior of the sample also has curvature and there are loose voids in the whole structure.

Inspired by theoretical predictions, curved carbon nanospheres with abundant curved surfaces were synthesized (**Figure** **2**a). Surfactants, commonly employed as soft templates, not only facilitate the formation of carbon matrices with diverse pore structures but also promote surface curvature during the pyrolysis process.^[^ ^8^, ^10^ ^]^ By employing Pluronic F127, a carbon sphere featuring abundant concave surfaces within the uniformly oriented mesochannels was obtained^[^ ^11^ ^]^ (Figure 2b). Interestingly, with the assistance of shearing flow to drive the spiral self‐assembly, a chiral multi‐shelled carbon hollow nanosphere was achieved by using Pluronic P123 as the soft template (Figure 2c). Its spiral architecture promotes the orientation of the carbon layers during the pyrolysis process. Normally, the TEM images are only 2D projection of 3D object, thus, electron tomography techniques were conducted to reveal the inner curved surfaces in real 3D. Figure 2b,c; Figures S3, S4, and Videos S1–S6 (Supporting Information) present the 3D reconstructions of two carbon structures with distinct curved surfaces: both have spherical morphology, but either with mesochannels, or chirality. Orthoslices acquired from different orientations were displayed in Figure 2b,c; Figures S3, S4, and Videos S1, S2, S3, S4, S5, and S6 (Supporting Information), indicating the existence of pores or spiral structures throughout the nanospheres, which provide abundant curved surfaces to anchor single atom catalysts. Furthermore, in situ electron tomography was applied to track the changes after the electrocatalysis reactions on a single particle level. As displayed in Figure 2d, the CCNs still keep their spherical morphology with mesochannels without the aggregation of CCNs, except for a little shrunk in size happens after reaction. The same situation holds for the nanosphere with chirality (Figure 2e). The above results indicate that the curved surface in CCNs was quite stable.

**Figure 3:**
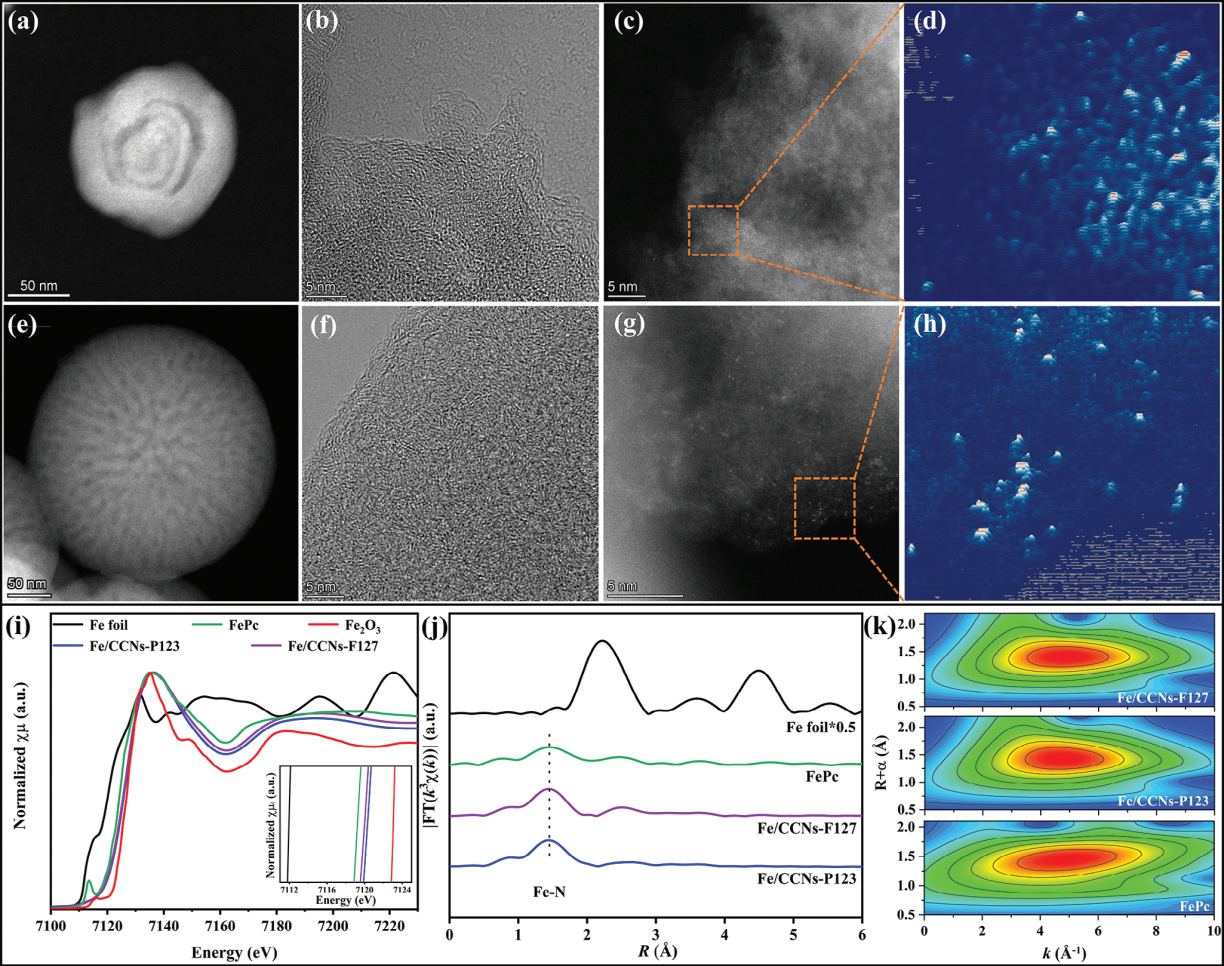
Structural characterizations of Fe/CCNs based on TEM and X‐ray absorption spectroscopy (XAS). a,b) High‐resolution TEM images, c) aberration‐corrected HAADF‐STEM image, and d) the partial enlarged HAADF‐STEM image of Fe/CCNs‐P123. e,f) High‐resolution TEM images, g) aberration‐corrected HAADF‐STEM image, and h) the partial enlarged HAADF‐STEM image of Fe/CCNs‐F127. i) Fe K‐edge XANES, j) *k* ^3^‐weighted FT‐EXAFS curves, and k) WT‐EXAFS spectra of the Fe/CCNs‐P123, Fe/CCNs‐F127 and the references.

After loading Fe with the help of NH_2_ groups decorated carbon quantum dots (NH_2_‐CQD), both the morphology and pore structure of the carbon matrices remain unchanged (**Figure** **3**a,e). Furthermore, the high‐resolution TEM images reveal that the carbon layers in the two matrices are distributed discontinuously and display geometrical bending at certain angles, providing further evidence for their intrinsic rich curved structure (Figure 3b,f). The high‐angle annular darkfield scanning transmission electron microscopy (HAADF‐STEM) images of both Fe/CCNs display plentiful monodispersed bright dots, revealing the atomic dispersion of isolated Fe atoms rather than nanoparticles or clusters (Figure 3c,d,g,h). The uniform distributions of Fe, N, O, and C were further confirmed by the corresponding energy dispersive X‐ray spectrum elemental mapping images (Figure S5, Supporting Information). Selected electron diffraction images show that Fe/CCNs‐P123 and Fe/ CCNs‐F127 have an amorphous nature and no aggregation of metal nanoparticles (Figure S6, Supporting Information).

Powder X‐ray diffraction (PXRD) was conducted to measure the iron and carbon crystalline structures of the as‐prepared samples. As shown in Figure S7 (Supporting Information), the PXRD results of the four samples displayed two diffraction peaks at about 24.5° and 43.3°, which are corresponding to (002) and (100)/(101) planes of graphitic carbon, respectively. Apart from the graphitic carbon peaks, the Fe/CCNs‐P123 and ‐F127 did not show any Fe nanoparticles (NPs) characteristic peaks, suggesting that the Fe species might be atomically dispersed.

To further investigate the specific surface areas and pore properties, nitrogen adsorption/desorption isotherms were measured at 77 K. As shown in Figure S8 (Supporting Information), the as‐prepared samples displayed a distinct uptake at low *P*/*P*_0_ of 0‐0.015 and a hysteresis loop at *P*/*P*_0_ of 0.4–0.95, suggesting their hierarchical microporous/mesoporous structures. After anchoring Fe atoms by secondary pyrolysis, Fe/CCNs‐P123 (760.2 m^2^ g^−1^) and ‐F127 (536.05 m^2^ g^−1^) exhibited a larger Brunauer–Emmett–Teller surface area (*S*_BET_) than CCNs‐P123 (640.29 m^2^ g^−1^) and ‐F127 (437.47 m^2^ g^−1^), respectively. This phenomenon was ascribed to the ordered transformation of unstable amorphous carbon from a disordered structure to a graphitic crystal structure during secondary pyrolysis. Such high surface areas and hierarchically porous structures of Fe/CCNs‐P123 and ‐F127 were regarded as beneficial for exposing the catalytic sites and improving the charge and mass transfer during ORR.

The carbon graphitization degrees of the CCNs matrices were also determined through Raman measurements, as shown in Figure S9 (Supporting Information). Raman spectra of the as‐prepared samples displayed two distinct peaks at about 1350 and 1580 cm^−1^ corresponding to the D band (defective carbon) and G band (graphitic carbon), respectively. Compared with those of CCNs‐P123 (1.05) and ‐F127 (1.03), the *I*_D_/*I*_G_ values of Fe/CCNs‐P123 (0.89) and ‐F127 (0.99) decreased, suggesting a slight increase of the graphitization degree, which could enhance the electrical conductivity and improve the electron transport during ORR processes.

X‐ray photoelectron spectroscopy (XPS) was carried out to study the surface chemical compositions of the as‐prepared samples. The full XPS survey spectra (Figure S10, Supporting Information) confirmed the existence of C, N, and O in all the samples, while the Fe/CCNs‐P123 and ‐F127 samples showed the presence of Fe species, suggesting the successful doping of Fe into the CCNs matrices. The Fe contents of Fe/CCNs‐P123 (2.76 wt%) and ‐F127 (2.94 wt%) were similar confirmed by inductively coupled plasma optical emission spectrometer (Table S1, Supporting Information). The high‐resolution N 1s spectra were mainly deconvoluted into three types N including the graphitic‐N (≈401 eV), pyridinic‐N (≈398 eV) and oxidized‐N (≈403 eV) (Figure S11, Supporting Information). The increased graphitic‐N contents in the Fe/CCNs‐P123 and ‐F127 samples indicated an improved graphitization degree of the CCN matrices, which was consistent with the Raman results. Notably, a high percentage of graphitic‐N species was favorable for a faster electron transfer to boost the electrocatalytic kinetics (Table S2, Supporting Information). More accurate electronic structures and local coordination information for Fe/CCNs‐P123 and ‐F127 were probed by synchrotron X‐ray absorption fine‐structure (XAFS) spectroscopy, including X‐ray absorption near‐edge structure (XANES) and extended X‐ray absorption fine structure (EXAFS) spectra. In the Fe K‐edge XANES spectra displayed in Figure 3i, the absorption edge positions of Fe/CCNs‐P123 and ‐F127 were located between those of FePc and Fe_2_O_3_, indicating that the average valence states of Fe were between +2 and +3. The Fourier transform (FT) *k* ^3^‐weighted EXAFS spectra of Fe/CCNs‐P123 and ‐F127 presented a primary peak at ≈1.5 Å, which originated from Fe─N scattering. Moreover, no Fe─Fe peak at ≈2.2 Å was observed, suggesting that the Fe atoms were atomically dispersed (Figure 3j and Figures S12 and S13, Supporting Information). Wavelet‐transformed (WT) EXAFS is a powerful informative technique for distinguishing backscattering atoms based on the atom masses due to the high resolutions in both *k* and *R* spaces. As illustrated in Figure 3k, the WT contour plots of Fe/CCNs‐P123 and ‐F127 exhibited only one intensity maximum at ≈4.8 Å^−1^, which was closer to that of Fe─N (about 4.7 Å^−1^) in FePc, further verifying the Fe─N coordination for Fe/CCNs‐P123 and ‐F127. The EXAFS fitting results revealed that the coordination number of Fe atom with N was ≈4 for Fe/CCNs‐P123 and ‐F127, as shown in Table S3 (Supporting Information), implying the existence of Fe─N_4_ coordination structures.

**Figure 4:**
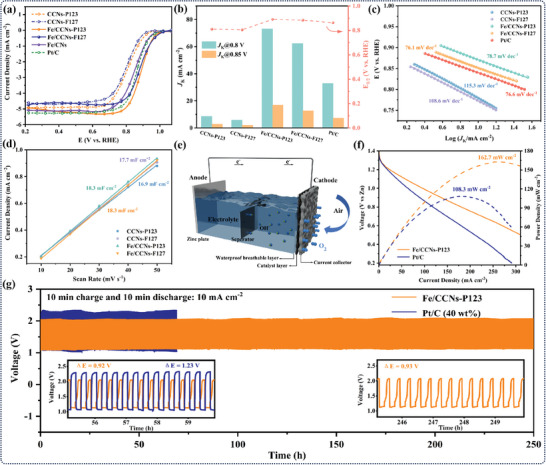
ORR and LZAB performance catalyzed by Fe/CCNs‐P123. a) LSV curves and b) *E*_1/2_ and *J*_k_ values for various catalysts in 0.1 m KOH. c) Tafel plots and d) *C*_dl_ values of the catalysts. e) Schematic configuration of the homemade LZAB. f) Discharge polarization curves and the corresponding power density curves of the LZABs using Fe/CCNs‐P123 and Pt/C as air electrodes, respectively. g) Galvanostatic discharge–charge cycle profiles of the LZABs.

The ORR catalytic activities of the as‐prepared samples were first examined by rotating disk electrode in an O_2_‐saturated 0.1 m KOH electrolyte. All the potentials reported were versus reversible hydrogen electrode. The cyclic voltammetry (CV) curves of the as‐prepared samples exhibited a prominent reduction peak in the O_2_‐saturated solution compared with the N_2_‐saturated solution, indicating their intrinsic ORR electrocatalytic activity (Figure S14, Supporting Information). Through linear sweep voltammetry (LSV) test (**Figure** **4**a), both Fe/CCNs‐P123 and ‐F127 exhibited superior ORR activity with a half‐wave potential (*E*_1/2_) of 0.89 and 0.884 V, respectively, which are better than that of the curvature‐free Fe/CNs (*E*_onset_ = 1.00 V, *E*_1/2_ = 0.85 V), indicating that curvature exerts a beneficial effect on the ORR performance (Figure S15, Supporting Information). Furthermore, the results also surpassed than those of commercial Pt/C (40 wt%, *E*_onset_ = 0.93 V, *E*_1/2_ = 0.86 V) and most of the catalysts with curvature (Table S4, Supporting Information). Moreover, the calculated kinetic current densities (*J*_K_) of Fe/CCNs‐P123 and ‐F127 at 0.85 V were 16.86 and 12.71 mA cm^−2^, respectively, which were higher than that of Pt/C (7.31 mA cm^−2^), demonstrating their excellent intrinsic activity (Figure 4b). The metal‐free CCNs‐P123 and ‐F127 matrices showed poor ORR activities with the *E*_1/2_ of 0.83 and 0.81 V, respectively, indicating the crucial role of the isolated Fe─N─C sites as the real ORR reaction sites. This result was further confirmed by the SCN^−^ poisoning experiment (Figure S16, Supporting Information), in which the *E*_1/2_ of Fe/CCNs‐P123 and ‐F127 displayed negative shifts after adding SCN^−^ due to their blocking effect on Fe sites.

In general, a small Tafel slope would reflect accelerated ORR kinetics. Compared to CCNs‐P123 (115.3 mV dec^−1^) and CCNs‐F127 (108.6 mV dec^−1^), both Fe/CCNs‐P123 (78.7 mV dec^−1^) and ‐F127 (76.1 mV dec^−1^) displayed small Tafel slopes, indicating that Fe─N─C sites could accelerate the kinetic process of ORR. Meanwhile, Fe/CCNs‐P123 and ‐F127 had a very similar Tafel slope to that of commercial Pt/C (40 wt%, 76.6 mV dec^−1^), suggesting that they were controlled by the same kinetic rate‐determining step (Figure 4c). The electron‐transfer kinetics of the Fe/CCNs catalysts toward ORR were further investigated by measuring LSV curves at various rotation speeds from 400 to 2500 rpm in an O_2_‐saturated solution. As shown in the insets of Figure S17 (Supporting Information), the electron transfer number (*n*) of Fe/CCNs‐P123 and ‐F127 based on Koutecky–Levich (K–L) were 3.90 and 3.97, respectively, which were close to the optimal value of 4.0, confirming dominant four‐electron ORR process.

In situ attenuated total reflection surface‐enhanced infrared reflection‐absorption spectroscopy, which can sensitively recognize the reaction intermediates and identify the reaction pathways, was conducted under ORR operating conditions. As illustrated in Figure S18 (Supporting Information), a prominent absorption band at ≈1220 cm^−1^ attributed to the antisymmetric bending mode of surface‐adsorbed *OOH was observed for Fe/CCNs‐P123 and ‐F127. As the potential decreased, the intensity of *OOH continued to increase, while the surface‐adsorbed *HOOH band at ≈1380 cm^−1^ was not identifiable across the entire potential range,^[^ ^12^ ^]^ further indicating that the four‐electron ORR process occurred on both Fe/CCNs‐P123 and ‐F127.

The electrochemical surface area (ECSA) of the as‐prepared samples was assessed by the double layer capacitance (*C*_dl_) in the non‐Faraday interval (Figure 4d and Figure S19, Supporting Information). Both Fe/CCNs and Fe‐free CCN matrices revealed similar *C*_dl_ values, which was indicative of their similar ECSAs. Therefore, the increase in ORR activity after Fe doping was attributed to the enhancement of the intrinsic activity rather than an enlarged active surface area. Electrochemical impedance spectroscopy measurements, as displayed in Figure S20 (Supporting Information) showed that Fe/CCNs had much smaller semicircle diameters than those of the metal‐free CCN matrices, which confirmed the decreased charge transfer resistance of Fe/CCNs catalysts, further suggesting the enhanced reaction kinetics by Fe doping.

The durability and methanol tolerance are the crucial parameters for practical electrocatalysts. Remarkably, Fe/CCNs‐P123 exhibits superior long‐term stability with no obvious decay in *E*_1/2_ after the 10 000^th^ potential cycles. In comparison, the *E*_1/2_ of Fe/CCNs‐F127 and Pt/C negatively shifted by 7 and 21 mV, respectively, after 10 000^th^ potential cycles (Figure S21a–c, Supporting Information). Furthermore, the excellent stability of Fe/CCNs‐P123 was also confirmed by the long‐term chronoamperometry measurement (*i–t*). After 20 h of continuous electrolysis, Fe/CCNs‐P123 still showed a high retention efficiency of 89.1%, whereas the retention efficiency of Fe/CCNs‐F127 decreased to 79.9%. In contrast, the Pt/C catalyst exhibited a remarkable decrease to 77.78% at 5 h (Figure S21d, Supporting Information). This finding indicated that the Fe/CCNs‐P123 catalyst had a very stable long‐life performance in ORR process. Moreover, Fe/CCNs‐P123 and ‐F127 exhibited outstanding methanol tolerance with slight changes in current density after methanol injection (5 mL), while a dramatic decay in the current density was occurred for commercial Pt/C (40 wt%) under identical conditions (Figure S22, Supporting Information). In addition, the PXRD pattern and HRTEM images demonstrated that the atomic dispersion of Fe atoms was still maintained, implying the structural robustness and outstanding stability of Fe/CCNs in the ORR process (Figures S23 and S24, Supporting Information).

The outstanding ORR performance of Fe/CCNs‐P123 promoted further exploration of its potential as an air cathode in rechargeable liquid zinc–air batteries (LZABs) (Figure 4e). The LZAB with a cathode composed of Fe/CCNs‐P123 exhibited a stable open circuit voltage (OCV) of 1.489 V (Figure S25, Supporting Information). In addition, the discharge polarization and power density curves of Fe/CCNs‐P123 and Pt/C (40 wt%)‐based LZABs were measured and displayed in Figure 4f. The peak power density of Fe/CCNs‐P123‐based LZAB reached to 162.7 mW cm^−2^ at 262.3 mA cm^−2^, surpassing that of the Pt/C (40 wt%)‐based one (108.3 mW cm^−2^ at 182.6 mA cm^−2^). Furthermore, galvanostatic cycling tests were performed at 10 mA cm^−2^ with 10 min of charge and 10 min of discharge to clarify the durability of the Fe/CCNs‐P123 and Pt/C (40 wt%)‐based LZAB. As depicted in Figure 4g, the Fe/CCNs‐P123‐based LZAB exhibited stable operation for 250 h without obvious voltage gap changes, whereas the Pt/C (40 wt%)‐based LZAB suffered serious degradation after 69 h. All the results proved the great potential of Fe/CCNs‐P123 catalyst for application in rechargeable LZABs.

**Figure 5:**
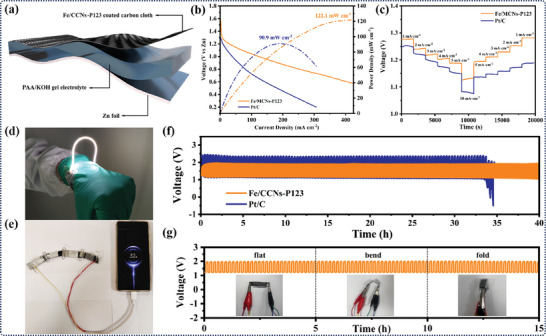
FZAB performances catalyzed by Fe/CCNs‐P123. a) Configuration of the flexible solid‐state zinc–air batteries. b) Discharging and power density plots of FZAB; c) Step discharge comparison diagrams at different current densities. d,e) The series‐connected FZABs can power a light strip (d) and charge a mobile phone (e). f,g) Galvanostatic discharge/charge cycling curves at 1 mA cm^−2^ of FZAB (f) under different bending angles (g).

To demonstrate practical applications in portable and wearable devices, a flexible solid‐state Zn–air battery (FZAB) was assembled using a polished Zn foil anode, polyacrylic acid/KOH–gel electrolyte, and a carbon cloth supported Fe/CCNs‐P123 cathode (**Figure** **5**a). The FZAB with a cathode composed of Fe/CCNs‐P123 exhibited a stable OCV of 1.28 V (Figure S26, Supporting Information). The peak power density of the flexible Fe/CCNs‐P123‐based FZAB was 122.1 mW cm^−2^ at 413.2 mA cm^−2^, surpassing that of the Pt/C (40 wt%)‐based one (90.9 mW cm^−2^ at 187.4 mA cm^−2^) (Figure 5b). From the discharge curves at different current densities, the flexible Fe/CCNs‐P123‐based FZAB exhibited higher voltages at all current densities compared to the Pt/C‐based one. When the current density resumed from 10 to 1 mA cm^−2^, the discharge voltage showed reversible recovery, indicating the outstanding rate performance and stability (Figure 5c). Three flexible Fe/CCNs‐P123‐based FZABs in series can light up a light‐emitting diode (3.0 V), while four such FZABs are capable of charging a mobile phone, revealing its potential applications in flexible electronics (Figure 5d,e). The cycling stability of both FZABs were further tested at a current density of 1 mA cm^−2^ with a 10‐min charge/10‐min discharge interval (Figure 5f). The Fe/CCNs‐P123 FZAB could supply a 1.95 V charge plateau and a 1.15 V discharge plateau, delivering a smaller voltage gap of 0.80 V compared to that of the Pt/C FZAB (1.23 V). After 40 h of continuous operation at 1 mA cm^−2^, the Fe/CCNs‐P123 FZAB showed a negligible deterioration in charging/discharging performance, while the Pt/C FZAB demonstrated poor performance under identical operating conditions with rapid degradation observed after just 33 h. The discharge–charge plateaus of Fe/CCNs‐P123‐based FZAB remains unchanged at different bending angles, indicating the remarkable flexibility of the Fe/CCNs‐P123‐based FZAB (Figure 5g).

## 3 Conclusion

In conclusion, DFT calculations clearly demonstrate that the curved surface, particularly the concave surface, can weaken the over‐strong adsorption of *OH and reduce the energy barriers of the RDS steps, thereby promoting the ORR kinetics. To demonstrate this concept, two CCN matrices with rich curved carbon surfaces were synthesized using a lamellar micelle spiral self‐assembly approach to prepare Fe SACs. Both Fe/CCNs catalysts display outstanding ORR performance with the *E*_1/2_ value of ≈0.89 V. Furthermore, practical applications of Fe/CCNs‐P123 in a FZAB exhibit a remarkable durability of 40 h with a high peak power density of 122.1 mW cm^−2^, and excellent charge–discharge performance at different bending angles. This study addresses the influence of surface curvature on promoting ORR performance, providing insights into the rational design of structural models and SAC catalysts with superior performance.

## Conflict of Interest

The authors declare no conflict of interest.

## Supporting information



Supporting Information

Supplemental Video 1

Supplemental Video 2

Supplemental Video 3

Supplemental Video 4

Supplemental Video 5

Supplemental Video 6

## Data Availability

The data that support the findings of this study are available from the corresponding author upon reasonable request.
