# Supplementary material for: In Situ Electron Tomography Insights into the Curvature Effect of a Concave Surface on Fe Single Atoms for Durable Oxygen Reaction
Source: Adv Sci (Weinh). 2024 Dec 16;12(6):2412387. doi: 10.1002/advs.202412387 (PMC11809330; doi:10.1002/advs.202412387)
Supplement: Supplementary file 1 — Supporting Information [file ADVS-12-2412387-s002.docx]

**Supporting Information**

**In Situ Electron Tomography Insights into the Curvature Effect of a Concave Surface on Fe Single Atoms for Durable Oxygen Reaction**

Jun-Kang Li,^[a]^ Haobo Zhao,^[b]^ Yang Zhang,^[c]^ Jing-Jing Ma,^[a]^ Fen-Fen Wang,^[a]^ Shuang-Quan Zang,*^[a]^ Jun Li,*^[b]^ and Shu-Na Zhao*^[a]^

**Experimental Section**

**1. Materials**

**Materials:** Pluronic P123 (PEO_20_PPO_70_PEO_20_) and Pluronic F127 (PEO_106_PPO_70_PEO_106_) were purchased from Sigma-Aldrich. Dopamine hydrochloride (C_8_H_11_NO_2_·HCl, DA), 1,3,5-trimethylbenzene (TMB), potassium persulfate (K_2_S_2_O_8_), ferrous chloride (FeCl_2_), dicyandiamide (DCDA), citric acid (CA), acrylic acid (AA) and N,N'-methylenebisacrylamide (MBA) were purchased from Shanghai Aladdin Reagent Co., Ltd. Potassium hydroxide (KOH) and ammonium hydroxide (NH_3_·H_2_O, 25-28 wt%) were purchased from Sinopharm Chemical Reagent Co., Ltd. Methanol, ethanol, and N,N-dimethylformamide (DMF) were purchased from Tianjin Kemiou Chemical Reagent Co., Ltd. Commercial Pt/C (40 wt%) was purchased from Johnson Matthey Co., Ltd. Nafion dispersion (5% w/w in water and 1-propanol) was supplied by Alfa Aesar. All reagents were obtained from commercial sources and used as received without further purification.

**2. Material characterizations**

Powder X-ray diffraction (PXRD) was tested on a Rigaku MiniFlex 600 diffractometer which using Cu Kα radiation (λ = 1.5418 Å) at 30 kV. The nitrogen adsorption/desorption isotherms were measured at liquid nitrogen temperature (77 K) with a Belsorp Max automatic volumetric adsorption system. The specific surface areas were analyzed based on Brunauer-Emmett-Teller (BET) theory. Pore size distribution data were calculated from the N_2_ sorption isotherms based on nonlocal density functional theory (NLDFT) model. The X-ray photoelectron spectroscopy (XPS) measurements were recorded on an AXIS SUPRA instrument with an Al Kα X-ray resource. Elemental analyses (EA) were carried out with a Perkin-Elmer 240 elemental analyzer. Inductively coupled plasma optical emission spectrometer (ICP-OES) was recorded with Shimadzu ICPE-9820. Raman spectra were obtained with a LabRam HR Evolution with excitation from the 532 nm line of an Ar ion laser. Transmission electron microscopy (TEM) was conducted using a FEI TalosF200s high-resolution transmission electron microscope with an accelerating voltage of 200 kV. EDS system: 2SDD windowless design, shutter-protected. Fast EDS mapping: pixel dwell times down to 10 μs. The high-angle annular dark-field transmission electron microscopy (HAADF-TEM) images were recorded by a JEOL JEM-ARM300F TEM/STEM with a spherical aberration corrector working at 300 kV. Fe K-edge analysis was performed with Si(111) crystal monochromators at the BL11B beamlines at the Shanghai Synchrotron Radiation Facility (SSRF) (Shanghai, China). Before the analysis at the beamline, samples were pressed into thin sheets with 1 cm in diameter and sealed using Kapton tape film. The X-ray absorption fine structure (XAFS) spectra were recorded at room temperature using a 4-channel Silicon Drift Detector (SDD) Bruker 5040. Fe K-edge extended XAFS (EXAFS) spectra were recorded in transmission mode. Negligible changes in the line-shape and peak position of Fe K-edge X-ray absorption near-edge structure (XANES) spectra were observed between two scans taken for a specific sample. The XAFS spectra of these standard samples (Fe foil, FePc, Fe_2_O_3_) were recorded in transmission mode. The spectra were processed and analyzed by the software codes Athena and Artemis.

**3. Experimental Section**

**Synthesis of CCNs-P123:**

Synthesis of CCNs-P123 was conducted according to the procedure reported by previous literature with a little modification^[1]^. Pluronic P123 (1.0 g) was first dissolved in a mixture of ethanol (50 mL) and water (50 mL), followed by adding dopamine (DA, 0.75 g) and 1,3,5-trimethylbenzene (TMB, 1.0 mL) into the above solution. The mixture was stirred at 300 rpm for 15 min to form a lamellar micelle system. Then, NH_3_·H_2_O (1.5 mL) was added to induce the polymerization of DA precursor. After continuously stirring at 300 rpm for 3 h, the mesostructured PDA nanospheres were collected by centrifugation, washed with ethanol and water several times, and freeze-dried for 24 h. After pyrolyzation first at 350 ℃ for 3 h and then at 900 ℃ for 2 h under a N_2_ atmosphere with a heating rate of 1.5 °C min^−1^, the spiral curved carbon nanospheres CCNs-P123 were obtained.

**Synthesis of CCNs-F127:** CCNs-F127 was synthesized using the same synthesis method, except that Pluronic P123 was replaced with Pluronic F127.

**Synthesis of** **NH_2_-CQD:**

NH_2_-CQD was prepared according to the previous report^[2]^. Citric acid (CA, 3.15 g, 15 mmol) and dicyandiamide (DCDA, 1.26 g, 15 mmol) were dissolved in deionized water (15 mL) with sonication and finally obtained a colorless and clear solution. The solution was then transferred to a hydrothermal autoclave reactor (50 mL) and heated at 180 °C for 6 h to obtain a brown-yellow solution. After naturally cooling to room temperature, the product was collected by centrifugation and washed with methanol three times, then dried at 60 ℃ to obtain brown-yellow powder as NH_2_-CQD.

**Synthesis of Fe/CCNs-P123 and Fe/CCNs-F127:**

The catalysts were prepared via the following procedure. First, CCNs-P123 (or CCNs-F127) (20 mg) was dispersed into ethanol (20 mL) with sonication. Then, 2 mL of NH_2_-CQD aqueous solution (1 mg·mL^−1^) and 43 μL of FeCl_2_ aqueous solution (0.02 mg·mL^−1^) were added into the above dispersion. The mixture was stirred at room temperature for 12 h, followed by removing the solvents under vacuum and drying in an oven. Finally, the obtained powder sample was calcined at 900 ℃ for 2 h under N_2_ atmosphere with a heating rate of 5 ℃ min^−1^ to afford Fe/CCNs-P123 (or Fe/CCNs-F127).

**4. Evaluation of electrochemical ORR performance:**

The electrochemical ORR measurements were carried out on a CHI 760E electrochemical workstation (Shanghai CH Instruments Co., Ltd., China) with a rotation-control equipment (Pine Instrument Company, USA). A glassy carbon electrode (GCE, diameter = 5 mm) loaded with catalyst was used as the working electrode. Pt wire and Hg/Hg_2_Cl_2_ (KCl-sat.) electrode were used as the counter electrode and reference electrode, respectively. The working electrode was prepared as follows: 1.0 mg of the as-synthesized catalyst was dispersed in 190 μL DMF and 10 μL Nafion (5 wt%) under sonication for at least 30 min to form a homogeneous ink. Then, 14 μL ink was loaded on a glassy carbon electrode with a mass loading of about 0.36 mg·cm^−2^, and then dried naturally at room temperature.

All the potentials reported were later converted to the RHE scale according to E (vs. RHE) = E (vs. Hg/Hg_2_Cl_2_) + 0.059 × pH + 0.241.

**5. Electrochemical measurements**

All the electrochemical experiments were conducted in O_2_-saturated or N_2_-saturated 0.1 M KOH solution at room temperature. Before the ORR measurement, the cyclic voltammetry (CV) experiments were performed from 0.2 - 1.2 V with a scan rate of 50 mV s^-1^ until a stable CV shape was obtained. Then the linear sweep voltammetry (LSV) experiments were performed at different rotation rates from 400 to 2500 rpm with a scan rate of 10 mV s^−1^. In the non-Faradaic potential region, CV curves at different scanning rates of 10-50 mV s^-1^ were executed to evaluate the double-layer capacitances (*C*_dl_) and electrochemical surface area (ECSA) of the catalysts. The tolerance toward methanol experiment was conducted by chronoamperometric response measurements at 0.7 V in O_2_-saturated 0.1 M KOH along with the injection of methanol (5 mL) at the time of 180 seconds. The poisoning experiment was conducted in O_2_-saturated 0.1 M KOH with the addition of 0.01 M KSCN. The accelerated durability tests (ADTs) were performed by applying potential cycling between 1.0 and 0.6 V for 5000 and 10000 cycles, respectively. Electrochemical impedance spectra (EIS) were conducted at potential 0.8 V, with amplitude of 5 mV and the frequency was from 0.1 Hz to 100 kHz.

The electron transfer number (*n*) was calculated by the Koutechy-Levich (K-L) equation:

$$\frac{1}{J}= \frac{1}{J_{L}}+ \frac{1}{J_{K}}= \frac{1}{{B\omega}^{1/2}}+ \frac{1}{J_{K}}$$

B = 0.62*nFC*_0_*D*_0_^2/3^*V*^-1/6^

Where *J*, *J_K_* and *J_L_* are the measured current density, kinetic and limiting current densities, respectively. *ω* is the angular velocity of the disk, *n* is the electron transfer number, *F* is the Faraday constant (96485 C·mol^-1^), *C*_0_ is the bulk concentration of O_2_ in KOH (1.2 × 10^-6^ mol·cm^-3^), *D*_0_ is the diffusion coefficient of O_2_ in 0.1 M KOH (1.9 × 10^-5^ cm^2^·s^-1^), and *V* is the kinematic viscosity of the electrolyte (0.01 cm^2^·s^-1^).

**6. Zn-air battery (ZAB) tests**

**6.1 Liquid Zn-air battery (LZAB) assembly and measurements**

In the assembly process of the LZAB, 3 mg of the as-prepared electrocatalyst was dispersed into a mixed solution including 100 µL ethanol, 80 µL H_2_O and 20 µL Nafion (5 wt%) with sonication. Then, 100 µL of the obtained ink was dropped onto a composite substrate material consisted of nickel foam, waterproof film, and carbon paper with a catalyst mass loading of 1.5 mg cm^-2^, which was used as the air cathode. A polished zinc plate was used as anode and 6 M KOH solution with 0.2 M zinc acetate as electrolyte.

The open circuit voltage and discharge polarization curve of the LZAB were measured with an electrochemical workstation CHI760E. The galvanostatic charge-discharge ability of the LZAB were tested using LAND-CT2001A.

**6.2 Flexible Zn-air battery (FZAB) assembly and measurements**

The PAA gel electrolyte was synthesized according to the literature. Dissolve 18 g of KOH in 26 g of deionized water, referred to as solution A. Prepare a polymer solution by adding 0.5 g of MBA to 3 g of AA solution and stir until dissolved, referred to as solution B. Dissolve 2 g of K_2_S_2_O_8_ in 10.5 g of deionized water, referred to as solution C. First, mix solution A with solution B and continue stirring for 5 minutes. Filter off the white precipitate formed during this process. Pour the remaining transparent solution into a petri dish. Finally, quickly drop solution C into the petri dish, and a gel will form quickly.

**7. Computation Details：**

Spin-polarized Density Functional Theory (DFT) calculations were calculated using the Vienna Ab initio Simulation Package (VASP)^[3]^. Using GGA-PBE^[4]^ functional and projector-augmented wave (PAW) pseudopotentials^[3, 5]^ to account for core-valence interactions. We carried out Kohn-Sham calculations with a kinetic energy cutoff of 450 eV for plane wave expansions. We optimized all the geometries using the limited-memory Broyden–Fletcher–Goldfarb–Shanno (LBFGS) algorithm in the Atomic Simulation Environment (ASE)^[6]^ and using VASP to calculate forces and energies. The convergence criteria are 0.02 eV/Å for the force and 10^-5^ eV for the energy. We used the implicit solvent model in VASPsol^[7, 8]^ for considering the solvent environment. To consider van der Waals interactions, we used the DFT-D3^[9]^ method with the Becke-Johnson damping function. For the Fe/MCNs-P123 model, we conducted calculations using 1×1×1 k-point sampling. For Fe/MCNs-F127 model, we used 3×3×1 k-point sampling. We calculated the electronic states and charge transfer through Bader charge analysis. We also used the VASPKIT code for post-processing of the VASP calculated data^[10]^.

For the oxygen reduction reaction (ORR), the Gibbs free energy change (ΔG) for each elemental step was calculated based on the computational hydrogen electrode (CHE) method by the following equation^[11]^:

ΔG = ΔE + ΔZPE - TΔS + ΔG_pH_ + ΔG_U_

where ΔE is the reaction energy of each step, obtained from DFT calculations; ΔZPE is the change of zero-point energies in the reactions; TΔS is the entropy contribution at 300 K; ΔG_pH_ is the correction of H^+^ concentration. ΔG_U_ is the influence of applied potential, defined as: ΔG_U_ = –eU, where U is the potential at the electrode.

**8. Supplementary Figures**


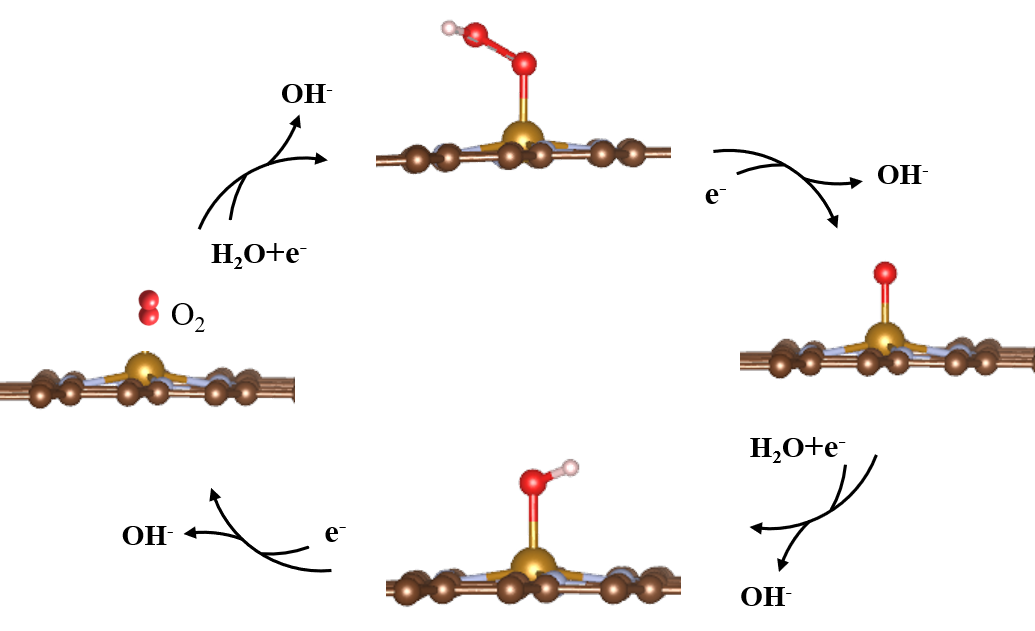


**Figure S1.** The reaction scheme with the intermediates in the ORR process on the planar surface of Fe/CCNs-P123.

**
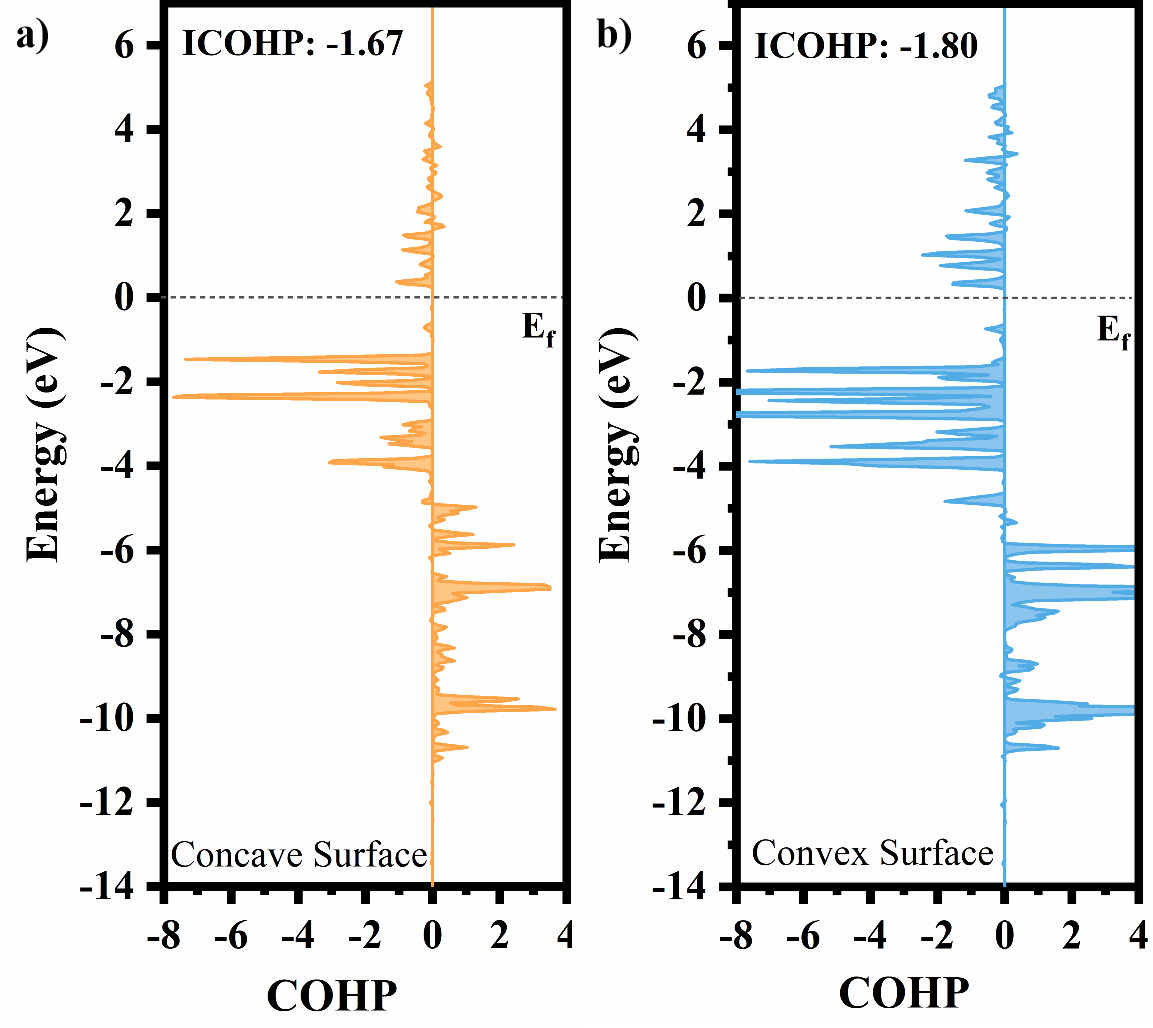
**

**Figure S2.** The COHP of concave surface (a) and convex surface (b) configuration after *OH adsorption.


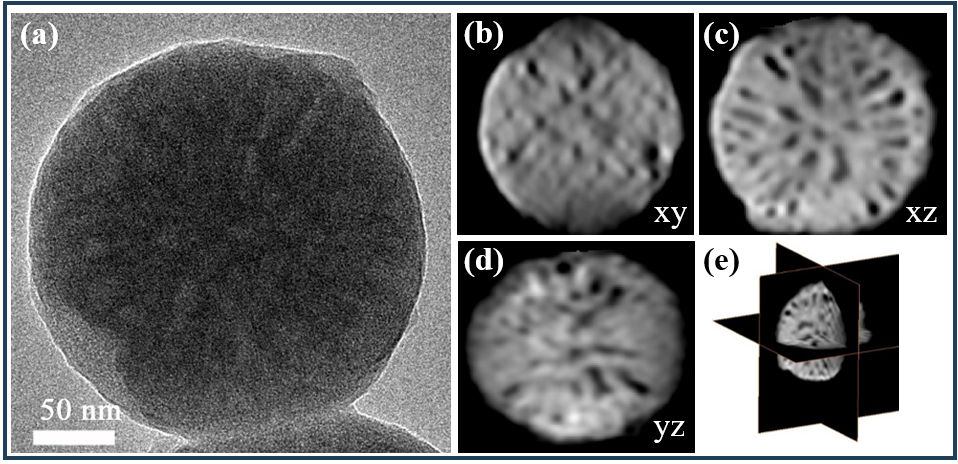


**Figure S3.** TEM image of the CCNs- F127 sample after electro-catalysis reaction. (b-d) Orthoslice from different direction of the 3D reconstructed volume. (e) Overview of the orthoslice.


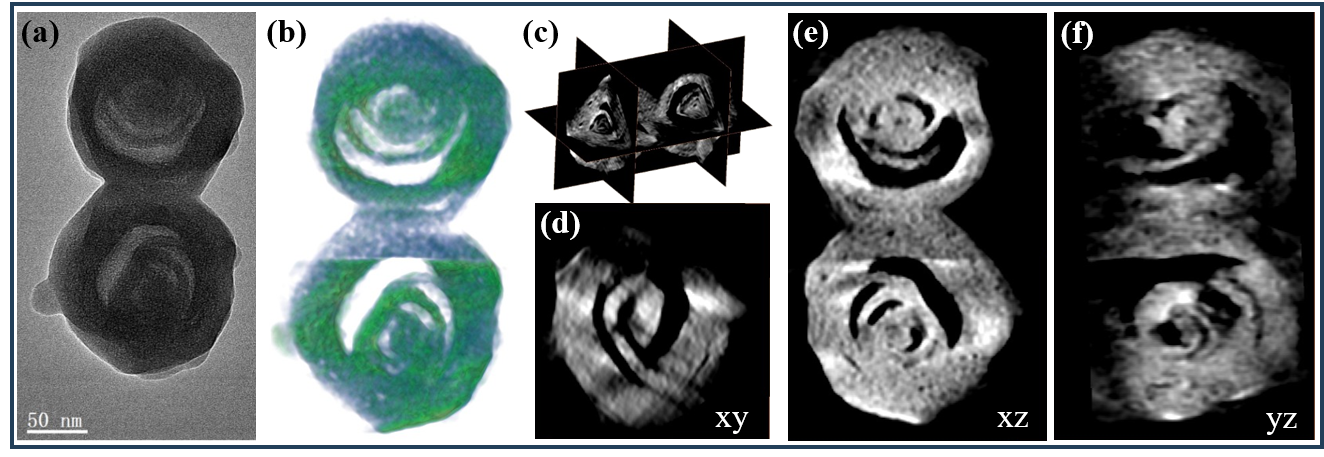


**Figure S4.** TEM image of the CCNs-P123 sample after electro-catalysis reaction. (b) Cross section view about the 3D reconstructed volume. (c) Overview of the orthoslice. (d-f) Orthoslice from different direction of the 3D reconstructed volume.

**
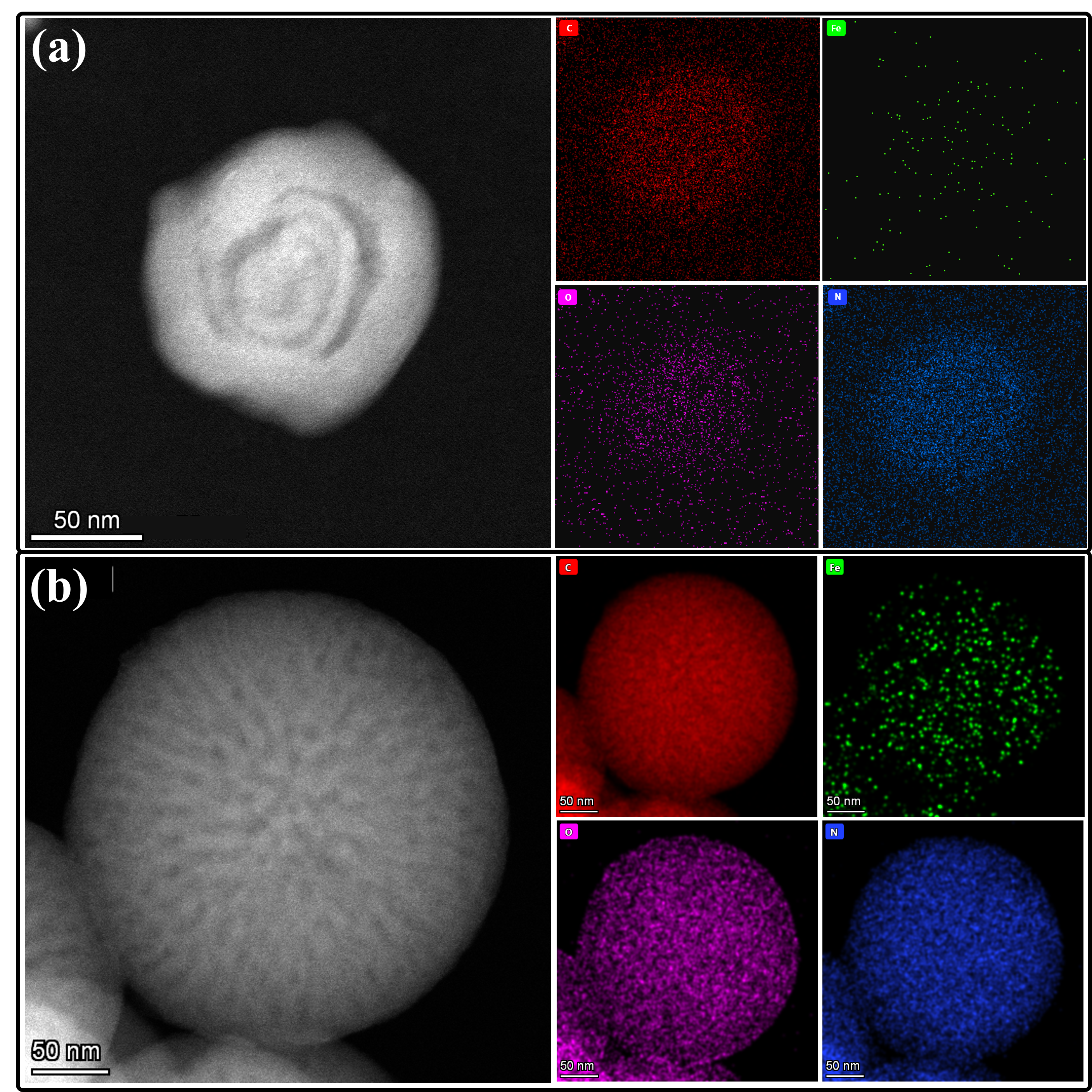
**

**Figure S5.** High-resolution TEM patterns and the corresponding elemental mapping of (a) Fe/CCNs-P123 and (b) Fe/CCNs-F127.


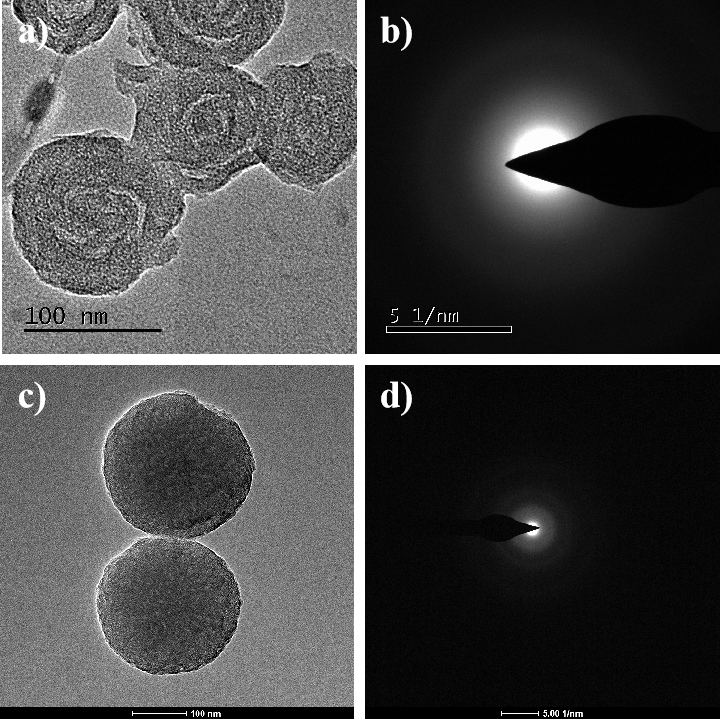


**Figure S6.** HRTEM images and the corresponding SAED patterns of the Fe/CCNs-P123 (a, b) and Fe/CCNs-F127 (c, d).


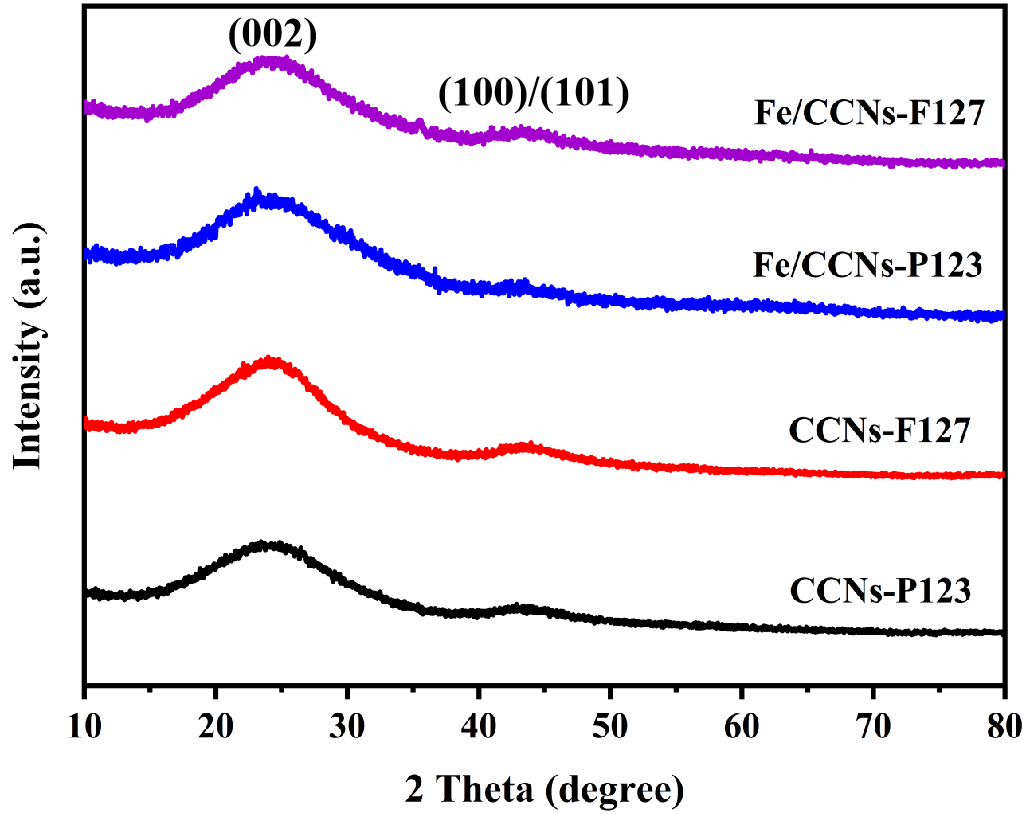


**Figure S7.** PXRD patterns of Fe/CCNs-F127, Fe/CCNs-P123, CCNs-F127, and CCNs-P123 samples, respectively.

**

**

**Figure S8**. N_2_ isothermal adsorption-desorption curves of catalysts.





**Figure S9.** Raman spectra of the as-prepared catalysts.





**Figure S10.** XPS survey spectra of Fe/CCNs-F127, Fe/CCNs-P123, CCNs-F127, and CCNs-P123 samples, respectively.





**Figure S11.** High-resolution XPS spectrum of N 1s for Fe/CCNs-F127, Fe/CCNs-P123, CCNs-F127, and CCNs-P123 samples.





**Figure S12.** FT-EXAFS fitting curve of Fe/CCNs-P123.





**Figure S13.** FT-EXAFS fitting curve of Fe/CCNs-F127.

**
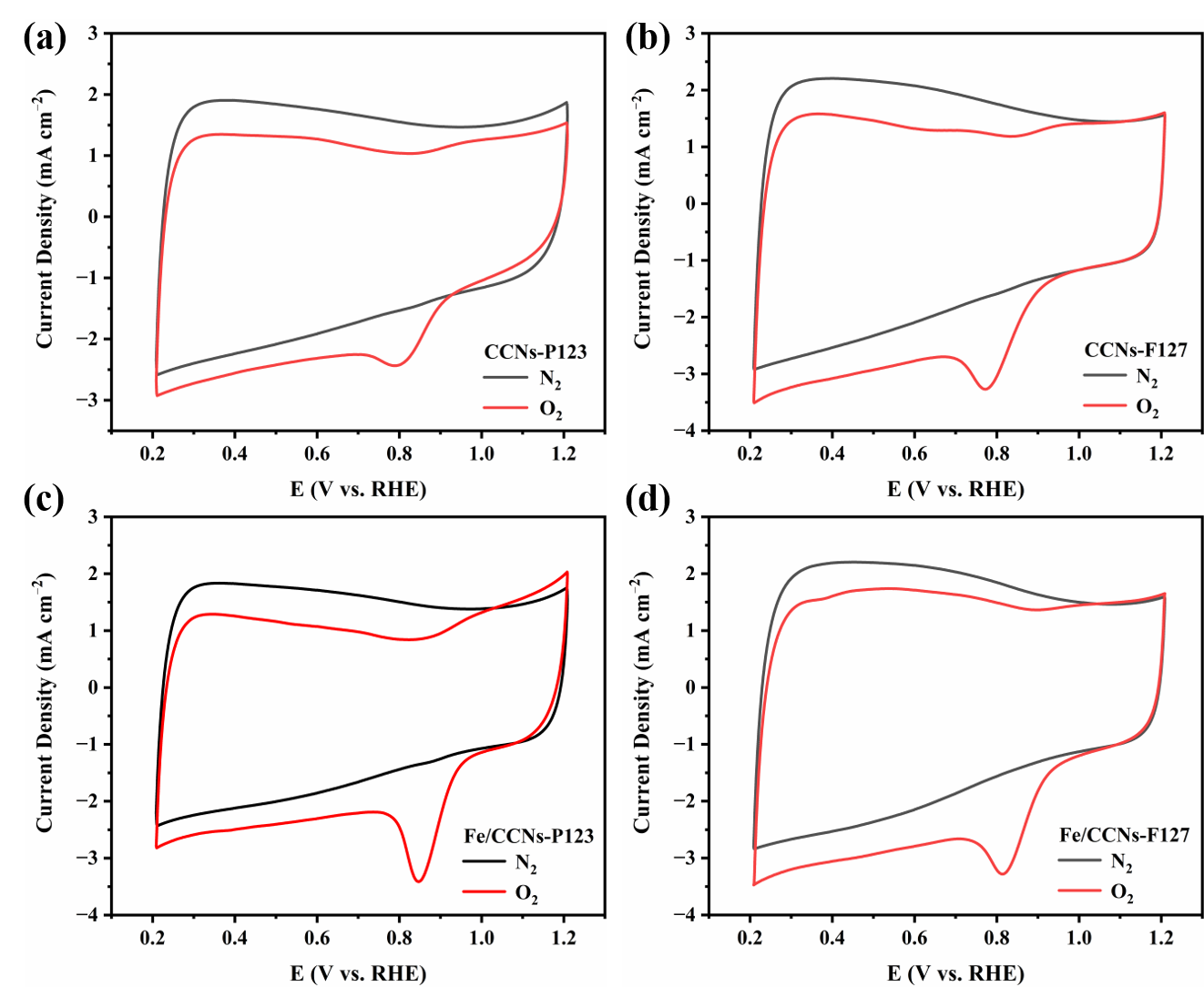
**

**Figure S14.** CV curves of the as-prepared samples (a) CCNs-P123, (b) CCNs-F127, (c) Fe/CCNs-P123, and (d) Fe/CCNs-F127 in O_2_- or N_2_- saturated 0.1 M KOH electrolyte.

**
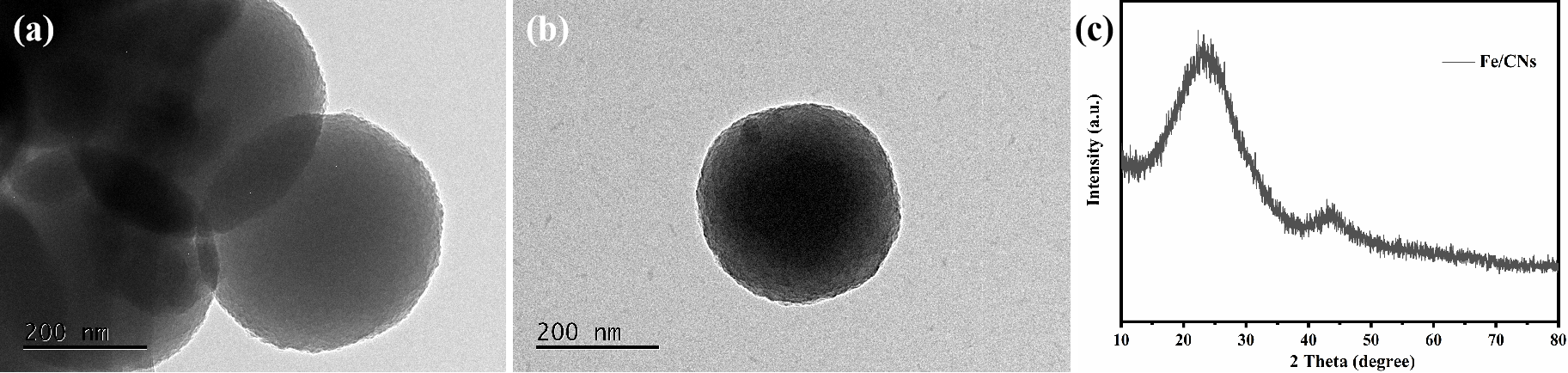
**

**Figure S15**. High-resolution TEM images of (a) CNs and (b) Fe/CNs. (c) PXRD pattern of Fe/CNs.

To ensure the comparability of the experimental results, the same lamellar micellar helical self-assembly strategy was employed to regulate the morphology of the carbon support by reducing the TMB/P123 mass ratio from 0.8 to 0.2. As the TMB/P123 mass ratio decreases from 0.8 to 0.2, the morphology of the resulting product transformed from high curvature multi-shell carbon nanospheres (CCNs) to curvature-free solid carbon nanospheres (CNs). After loading Fe species, the morphology of Fe/CNs remained unchanged. The PXRD result exhibited no diffraction peaks of Fe nanoparticles, indicating that the Fe species might be atomically dispersed.





**Figure S16.** LSV curves of Fe/CCNs-P123 and Fe/CCNs-F127 in O_2_-saturated 0.1 M KOH solution before and after addition of KSCN into the solution.


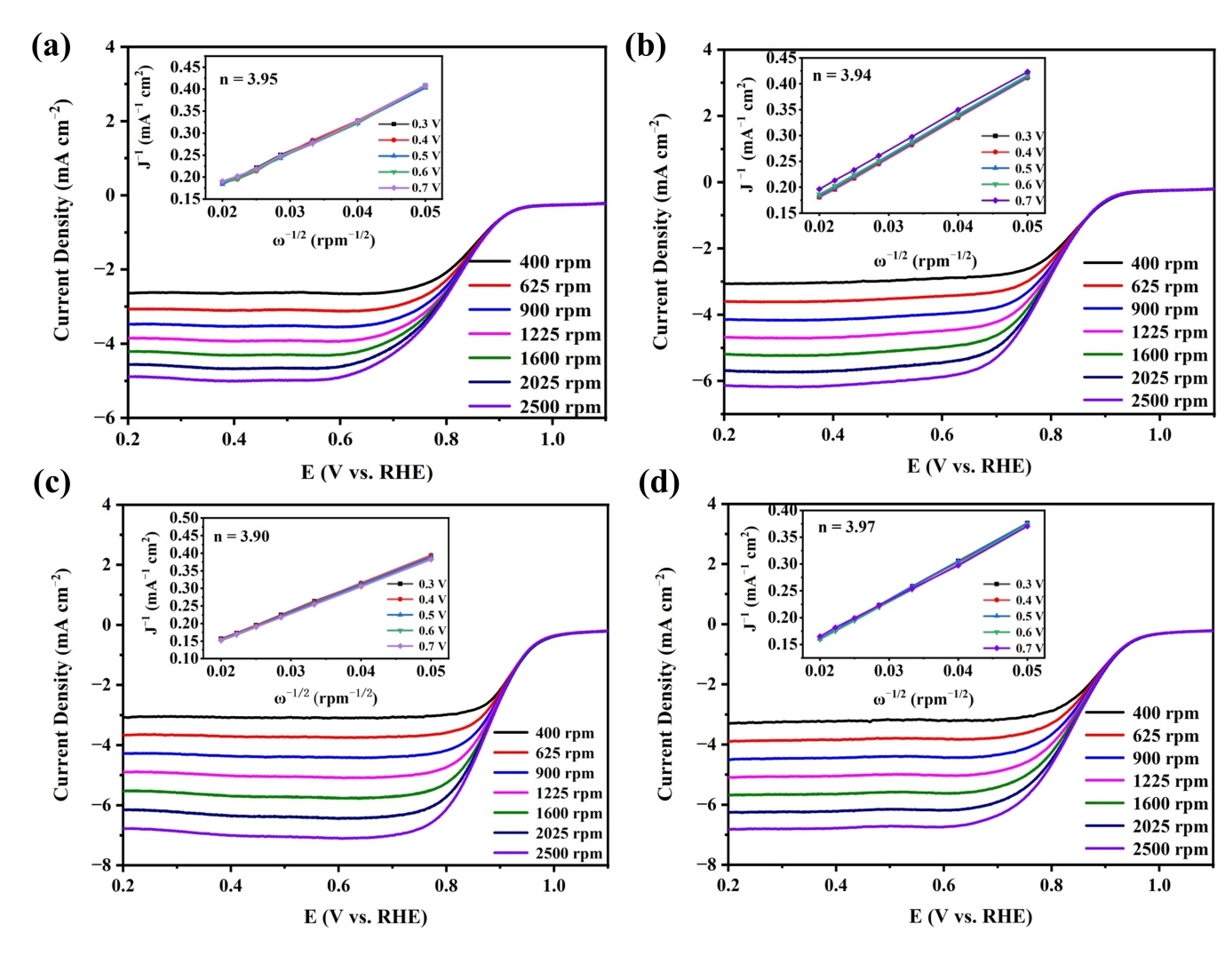


**Figure S17.** The ORR polarization curves of (a) CCNs-P123, (b) CCNs-F127, (c) Fe/CCNs-P123, and (d) Fe/CCNs-F127 at different rotation speeds. Insets: Koutecky-Levich plots and electron transfer number (*n*).





**Figure S18.** *In situ* ATR-FTIR spectra toward ORR over Fe/CCNs-P123 and Fe/CCNs-F127 at the applied potential ranging from 0.8 to 0 V vs RHE.

**
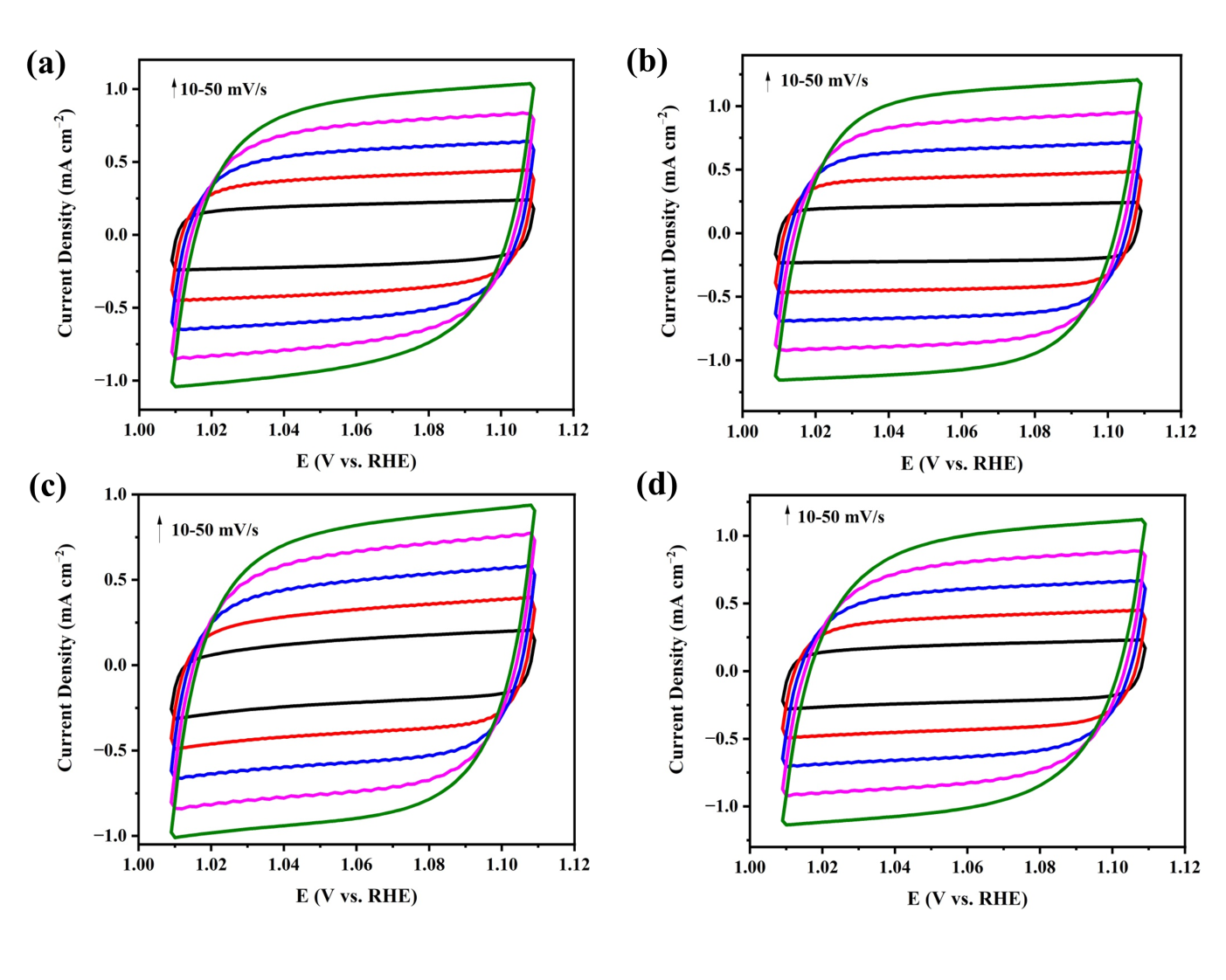
**

**Figure S19.** CV curves of (a) CCNs-P123, (b) CCNs-F127, (c) Fe/CCNs-P123, and (d) Fe/CCNs-F127, which were recorded in 0.1 M KOH solution with different scan rates from 10 to 50 mV·s^-1^.





**Figure S20.** EIS plots of as-prepared samples.

**
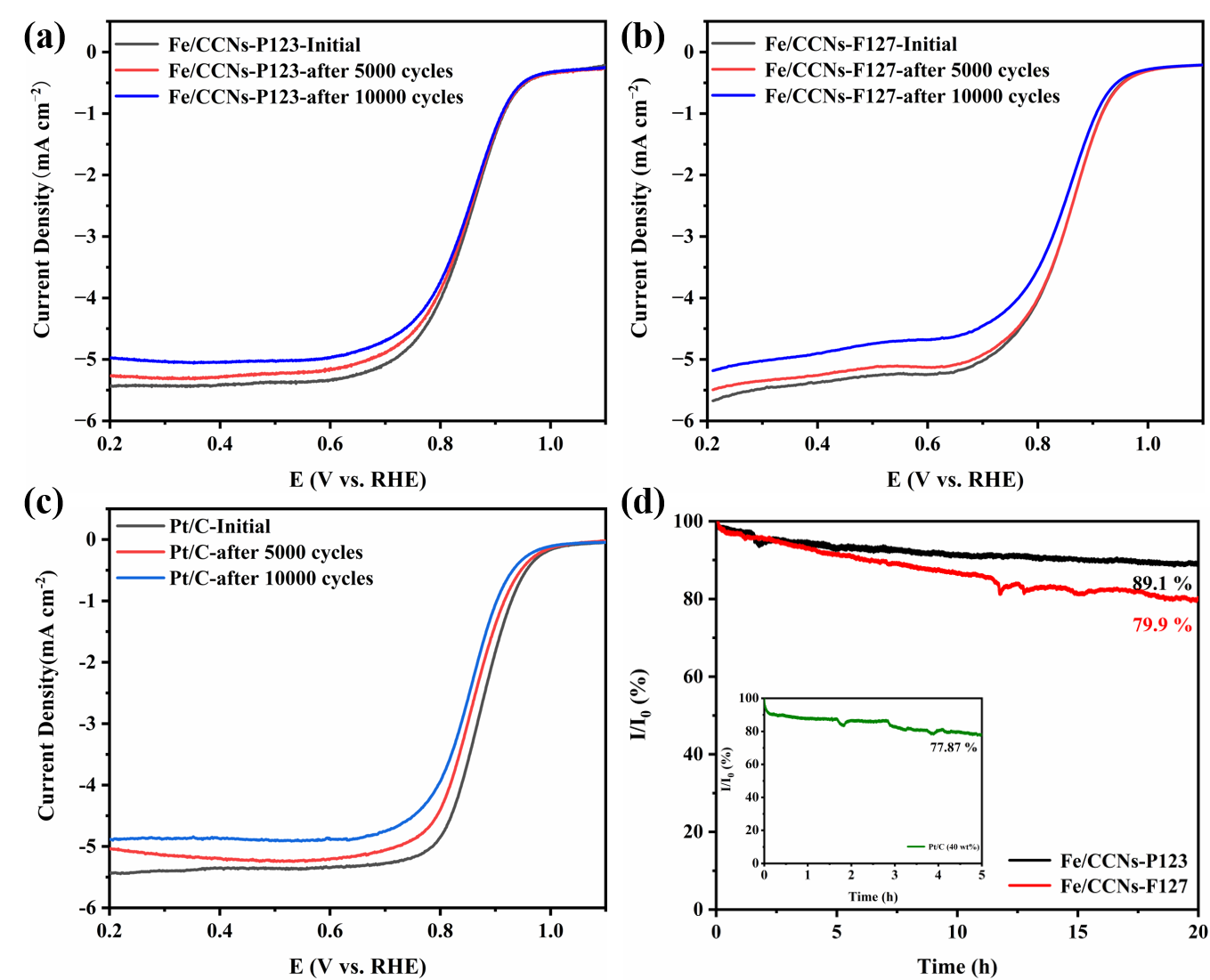
**

**Figure S21.** ORR polarization curves of (a) Fe/CCNs-P123 (b) Fe/CCNs-F127 (c) Pt/C before and after 5000 and 10000 cycles. (d) Chronoamperometric response of Fe/CCNs-P123, Fe/CCNs-F127 and Pt/C catalysts.

**

**

**Figure S22.** (a) Methanol crossover effect of Fe/CCNs-P123, Fe/CCNs-F127 and Pt/C catalysts at 0.7 V with injection of 5 mL methanol.


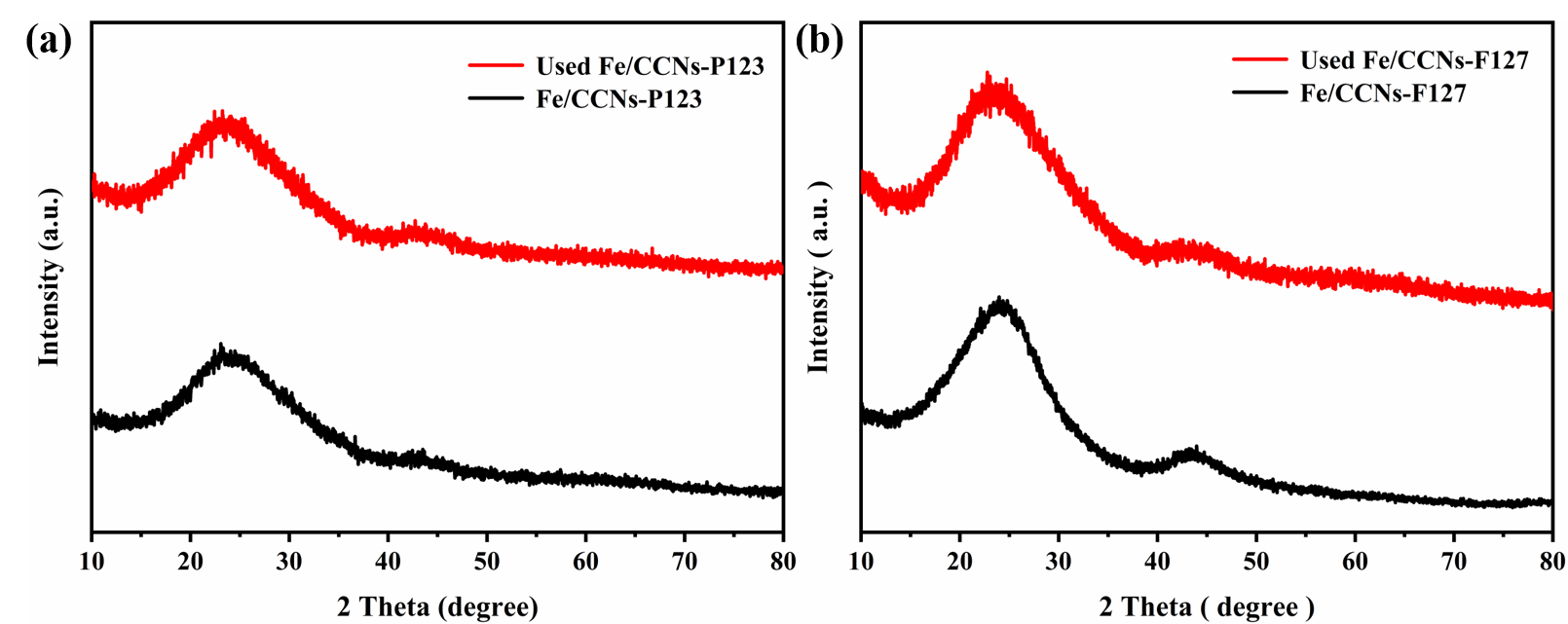


**Figure S23.** PXRD patterns of (a) Fe/CCNs-P123 and (b) Fe/CCNs-F127 after accelerating durability tests.


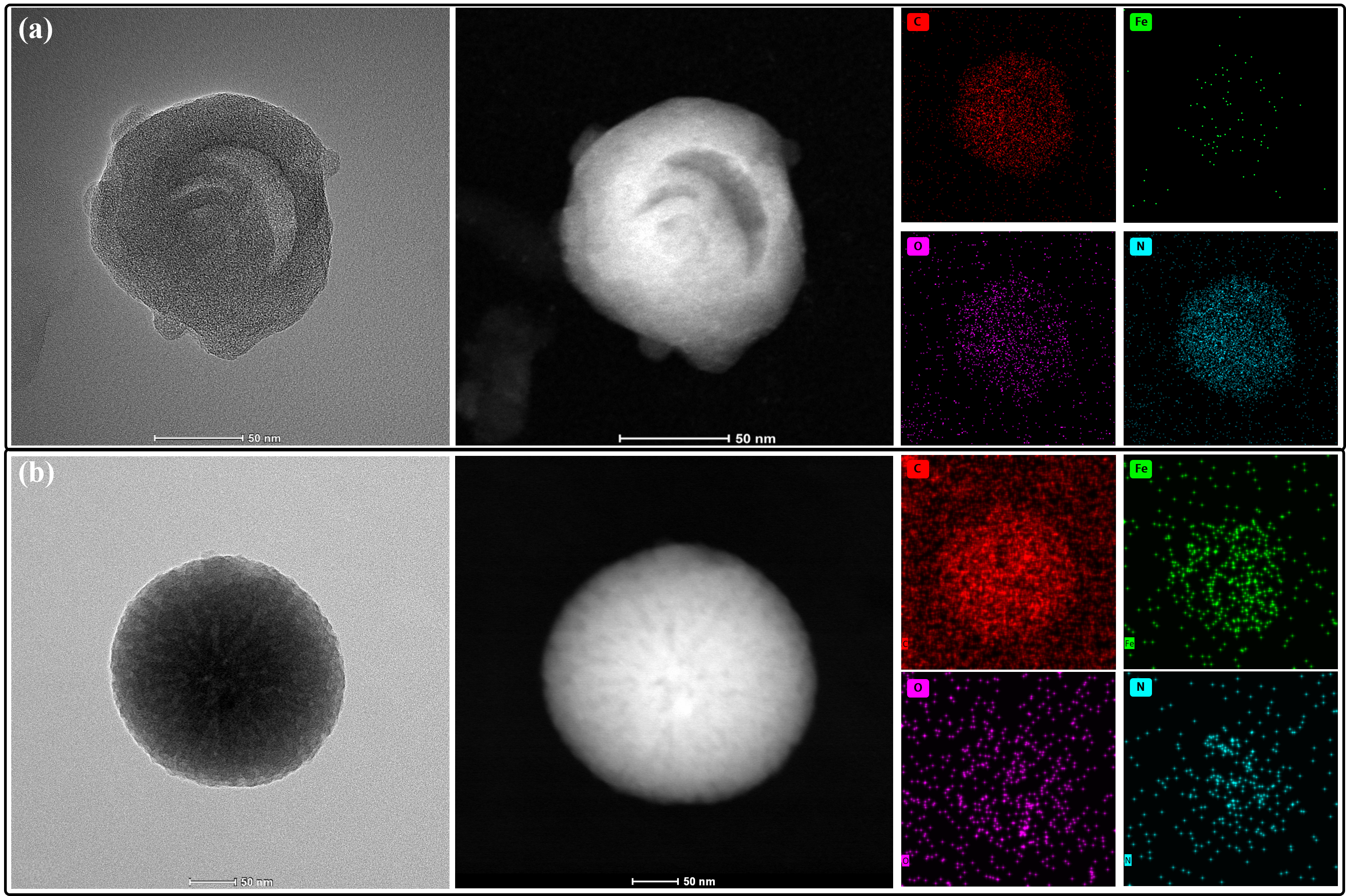


**Figure S24.** High-resolution TEM patterns and the corresponding elemental mapping of (a) Fe/CCNs-P123 and (b) Fe/CCNs-F127 after accelerating durability tests.


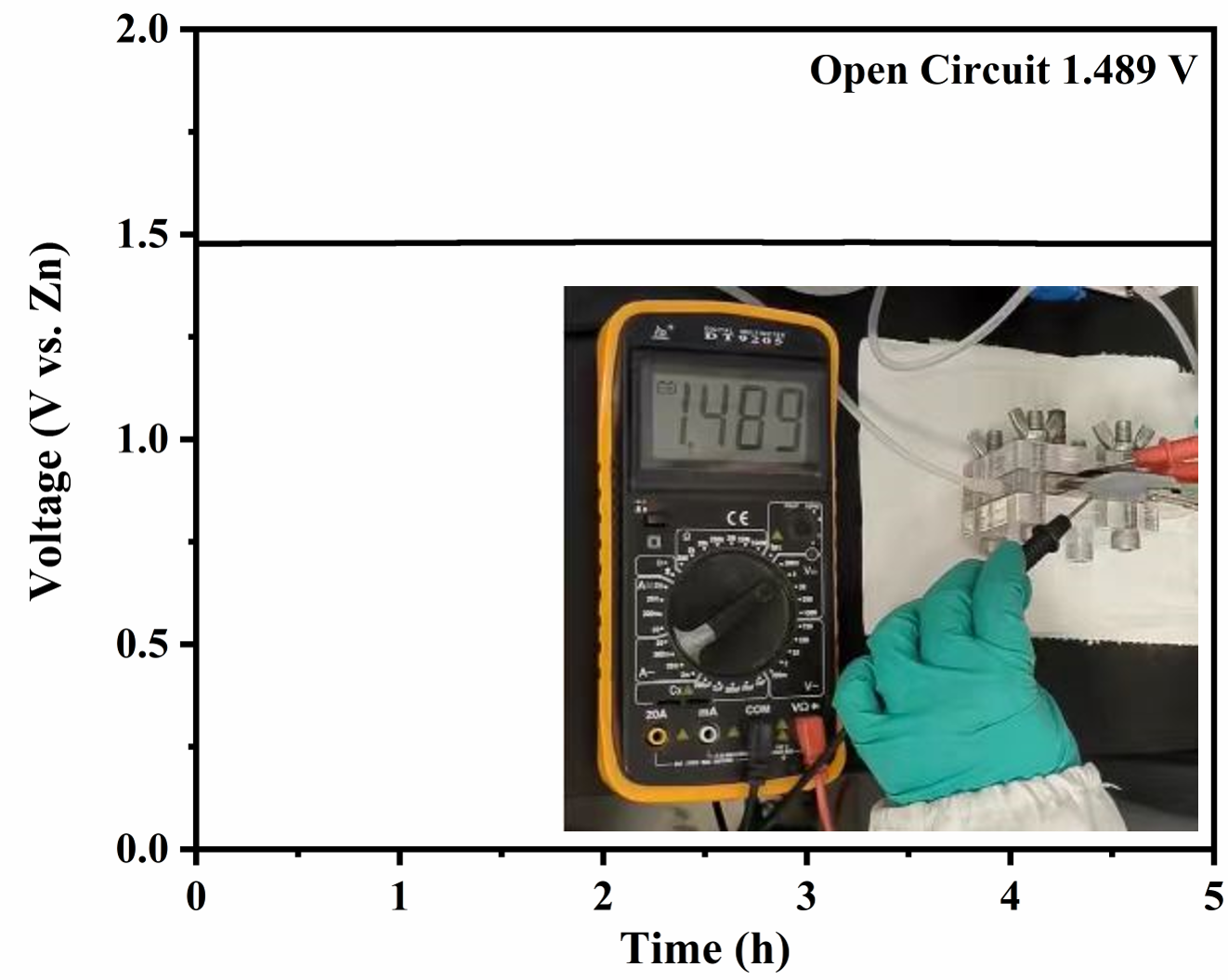


**Figure S25.** Electrochemical performance of LZABs using Fe/CCNs-P123. Open circuit voltage vs. time curve of Fe/CCNs-P123.


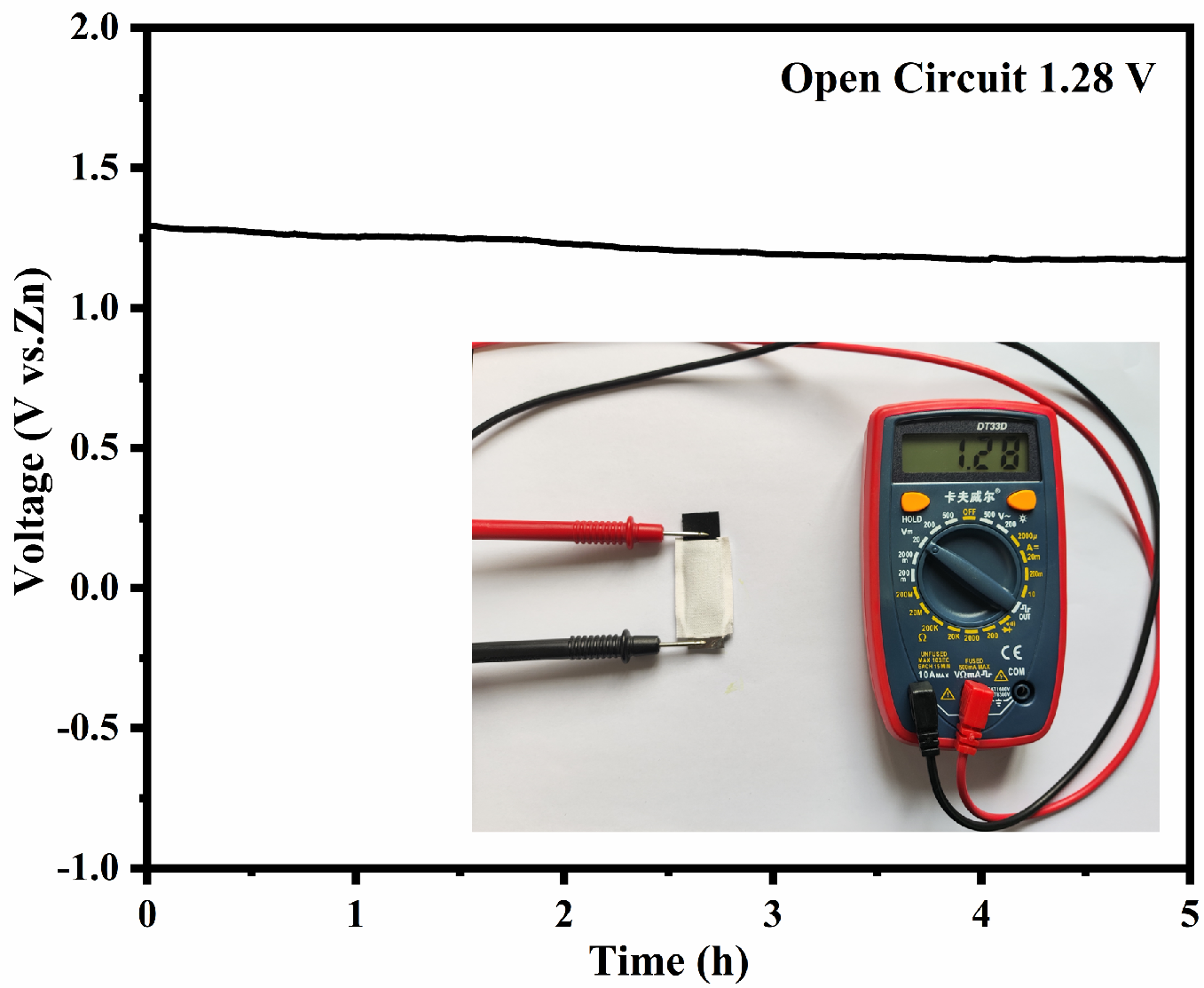


**Figure S26.** Electrochemical performance of FZABs using Fe/CCNs-P123. Open circuit voltage vs. time curve of Fe/CCNs-P123.

**Table S1.** EA and Fe contents measured by ICP-OES for the as-prepared catalysts.

| Sample | EA | | | | ICP-OES |
| --- | --- | --- | --- | --- | --- |
|  | N (wt%) | C (wt%) | H (wt%) | O(wt%) | Fe Loading(wt%) |
| CCNs-P123 | 4.27 | 76.39 | 1.22 | 10.44 | - |
| CCNs-F127 | 5.12 | 76.57 | 1.03 | 7.79 | - |
| Fe/CCNs-P123 | 1.53 | 58.00 | 2.40 | 9.14 | 2.76 |
| Fe/CCNs-F127 | 2.26 | 63.90 | 1.78 | 12.37 | 2.94 |

**Table S2.** The relative contents of different N-sites derived from high-resolution XPS scans of N 1s. for the as-prepared catalysts.

| Sample | Graphitic-N (at %) | Pyridinic-N (at %) | Oxidized-N (at %) |
| --- | --- | --- | --- |
| CCNs-P123 | 52.48 | 37.28 | 10.24 |
| CCNs-F127 | 48.51 | 38.67 | 12.82 |
| Fe/CCNs-P123 | 59.1 | 30.83 | 10.07 |
| Fe/CCNs-F127 | 59.03 | 28.03 | 12.94 |

**Table S3.** Summary of FT-EXAFS results.

| Sample | Path | CN | R (Å) | σ^2^ (10^-3^Å^2^) | *∆E*_0_ (eV) | R-factor |
| --- | --- | --- | --- | --- | --- | --- |
| Fe foil | Fe-Fe1 | 8* | 2.47±0.01 | 5.0±1.4 | 6.6±2.2 | 0.003 |
|  | Fe-Fe2 | 6* | 2.85±0.01 | 6.3±2.5 | 6.6±2.2 |  |
| Fe/CCNs-P123 | Fe-N/O | 3.9±0.3 | 1.95±0.01 | 9.1±1.4 | -6.3±1.9 | 0.011 |
| Fe/CCNs-F127 | Fe-N/O | 3.8±0.6 | 1.93±0.01 | 8.6±1.7 | -7.6±2.1 | 0.007 |

CN is the coordination number; R is interatomic distance (the bond length between central atoms and surrounding coordination atoms); σ^2^ is Debye-Waller factor (a measure of thermal and static disorder in absorber-scatterer distances); *∆E*_0_ is edge-energy shift (the difference between the zero kinetic energy value of the sample and that of the theoretical model); R factor is used to value the goodness of the fitting. *S_0_^2^ is the amplitude reduction factor. *S_0_^2^ was fixed to 0.83 as determined from Fe foil fitting. *This value was fixed during EXAFS fitting, based on the known structure of Fe foil. The data range used for data fitting in *k*-space (*∆*k) and *R*-space (*∆*R) are 3.0-11 Å^−1^ and 1.0-2.2 Å, respectively.

**Table S4.** Comparison of the ORR activity of various curvature carbon matrices reported in the literature.

| Catalysts | *E*_onset_  (V_RHE_) | *E*_1/2_  (V_RHE_) | Mass loading (mg cm^-2^) | *J*_k_  (mA cm^-2^) | Electrolyte | Ref |
| --- | --- | --- | --- | --- | --- | --- |
| **Fe/CCNs-P123** | **1.05** | **0.89** | **0.34** | **16.86 @0.85 V** | **0.1 M KOH** | **This work** |
| **Fe/CCNs-F127** | **1.06** | **0.884** | **0.34** | **12.71**  **@0.85 V** | **0.1 M KOH** | **This work** |
| Co-N-C@HCNT | 0.90 | 0.86 | 0.2 | 8.44  @0.85 V | 0.1 M KOH | *Adv. Funct. Mater.* 2024, 2417621 |
| Fe-SAC-70 | / | 0.91 | 0.5 | 24.9  @0.85 V | 0.1 M KOH | [*Nat. Commun.*](https://www.x-mol.com/paper/journal/49) 2024, 15,1719 |
| L/Fe-NC | ~0.9 | 0.82 | 0.6 | / | 0.5 M H_2_SO_4_ | [*Chem. Eng. J.*](https://www.x-mol.com/paper/journal/144) 2023, 477, 146841 |
| FeN_4_-hcC | / | 0.85 | 0.6 | 3.87  @0.85 V | 0.5 M H_2_SO_4_ | *Adv. Mater.* 2023, 35, 2300907 |
| OLC/Co-N-C | / | 0.855 | 0.2 | / | 0.1 M  KOH | *Angew. Chem. Int. Ed.* 2021, 60, 12759 |
| FeSA-N/TC | / | 0.925 | 0.5 | 18.8  @0.9 V | 0.1 M KOH | *Angew. Chem. Int. Ed.* 2024, e202415691 |
|  | / | 0.825 | 0.75 | 11.4  @0.8 V | 0.1 M HClO_4_ |  |
| FeNS/Fe_3_C@CNS | 0.90 | 0.91 | / | / | 0.1 M KOH | *Angew. Chem. Int. Ed.* 2024, 136, e202313034 |
|  | 1.02 | 0.78 | / | / | 0.1 M HClO_4_ |  |
| B,N-Co/GCCN | ~0.97 | 0.82 | 0.51 | / | 0.1 M KOH | [*J. Alloys Compd.*](https://www.x-mol.com/paper/journal/430) 2022, 910, 164829 |
| metal/NG | / | 0.836 | 0.6 | / | 0.1 M HClO_4_ | [*ACS Mater. Lett.*](https://www.x-mol.com/paper/journal/1482) 2022, 4, 978 |
| Cu/CNT-8 | 0.933 | 0.863 | 0.4 | 9.24 @0.85 V | 0.1 M KOH | [*Nat. Commun.*](https://www.x-mol.com/paper/journal/49) 2021, 12, 6335 |
| PC | / | 0.89 | 0.5 | 3.76  @0.9 V | 0.1 M KOH | [*Int. J. Hydrogen Energy*](https://www.x-mol.com/paper/journal/579) 2024, 80, 788 |
| PdNiRu NSs | 1.016 | 0.898 | 0.08 | 5.52  @0.9 V | 0.1 M KOH | [*Nano Res.*](https://www.x-mol.com/paper/journal/68) 2024, 17, 3777 |
| P-FeNi-NPC | / | 0.845 | 0.4 | 9.01  @ 0.8 V | 0.1 M KOH | *Small* 2024, 2402762 |
| HCHDC | 1.00 | 0.88 | 0.6 | 22.5  @0.8 V | 0.1 M KOH | *Small* 2024, 20, 2401447 |
| T-Fe SAC | / | 0.91 | 0.2 | 28.7  @0.85 V | 0.1 M KOH | *Angew. Chem. Int. Ed.* 2024, e202319370 |
| S-Zn-N-C-950 | 1.01 | 0.89 | / | / | 0.1 M KOH | *Adv. Funct. Mater.* 2023, 2311337 |
| FeNCNT | 0.94 | 0.87 | 0.1 | / | 0.1 M KOH | [*J. Alloys Compd.*](https://www.x-mol.com/paper/journal/430) 2024, 984, 174002 |

**References**

[1] L. Peng, H. Peng, Y. Liu, X. Wang, C.-T. Hung, Z. Zhao, G. Chen, W. Li, L. Mai, D. Zhao, *Sci. Adv*. **2021**, *7*, eabi7403.

[2] J. Lin, J. Ding, H. Wang, X. Yang, X. Zheng, Z. Huang, W. Song, J. Ding, X. Han, W. Hu, *Adv. Mater.* **2022**, *34*, 2200559.

[3] G. Kresse, J. Furthmüller, *Phys. Rev. B* **1996**, *54*, 11169.

[4] J. P. Perdew, K. Burke, M. Ernzerhof, *Phys. Rev. Lett*. **1996**, *77*, 3865.

[5] G. Kresse, D. Joubert, *Phys. Rev. B* **1999**, *59*, 1758.

[6] A. H. Larsen, J. J. Mortensen, J. Blomqvist, I. E. Castelli, R. Christensen, M. Dułak, J. Friis, M. N. Groves, B. Hammer, C. Hargus, E. D. Hermes, P. C. Jennings, P. B. Jensen, J. Kermode, J. R. Kitchin, E. L. Kolsbjerg, J. Kubal, K. Kaasbjerg, S. Lysgaard, J. B. Maronsson, T. Maxson, T. Olsen, L. Pastewka, A. Peterson, C. Rostgaard, J. Schiøtz, O. Schütt, M. Strange, K. S. Thygesen, T. Vegge, L. Vilhelmsen, M. Walter, Z. Zeng, K. W. Jacobsen, *J. Phys.: Condens. Matter* **2017**, *29*, 273002.

[7] K. Mathew, V. S. C. Kolluru, S. Mula, S. N. Steinmann, R. G. Hennig, *J. Chem. Phys.* **2019**, *151*, 234101.

[8] K. Mathew, R. Sundararaman, K. Letchworth-Weaver, T. A. Arias, R. G. Hennig, *J. Chem. Phys*. **2014**, *140*, 084106.

[9] S. Grimme, S. Ehrlich, L. Goerigk, *J. Comput. Chem.* **2011**, *32*, 1456-1465.

[10] V. Wang, N. Xu, J.-C. Liu, G. Tang, W.-T. Geng, *Comput. Phys. Commun.* **2021**, *267*, 108033.

[11] J. K. Nørskov, J. Rossmeisl, A. Logadottir, L. Lindqvist, J. R. Kitchin, T. Bligaard, H. Jónsson, *J. Phys. Chem. B* **2004**, *108*, 17886-17892.
